# The impact of COVID-19 vaccination in prisons in England and Wales: a metapopulation model

**DOI:** 10.1186/s12889-022-13219-4

**Published:** 2022-05-18

**Authors:** Ciara V. McCarthy, Oscar O’Mara, Edwin van Leeuwen, CMMID COVID-19 Working Group, Katharine Sherratt, Kaja Abbas, Kerry LM Wong, Katherine E. Atkins, Rachel Lowe, Sophie R Meakin, Nicholas G. Davies, Timothy W Russell, Kathleen O’Reilly, Stéphane Hué, Emilie Finch, C Julian Villabona-Arenas, W John Edmunds, Yalda Jafari, Damien C Tully, Nikos I Bosse, Carl A B Pearson, David Hodgson, Adam J Kucharski, Graham Medley, Yang Liu, Simon R Procter, William Waites, Sam Abbott, Rosanna C Barnard, Fiona Yueqian Sun, Hamish P Gibbs, Rosalind M Eggo, Lloyd A C Chapman, Stefan Flasche, Akira Endo, Paul Mee, James D Munday, Mihaly Koltai, Amy Gimma, Christopher I Jarvis, Matthew Quaife, Samuel Clifford, Sebastian Funk, Kiesha Prem, Gwenan M Knight, Rachael Pung, Oliver Brady, Billy J Quilty, Mark Jit, Frank Sandmann

**Affiliations:** 1 Centre for Mathematical Modelling of Infectious Diseases, London School of Hygiene and Tropical Medicine, London, UK; 2 Her Majesty’s Prison and Probation Service, London, UK & the University of Nottingham, Nottingham, UK; 3 Statistics, Modelling and Economics Department, UK Health Security Agency, London, UK; 4 Current Address: European Centre for Disease Prevention and Control (ECDC), Stockholm, Sweden

**Keywords:** COVID-19, Vaccination, Prisons, Mathematical model, Public health

## Abstract

**Background:**

High incidence of cases and deaths due to coronavirus disease 2019 (COVID-19) have been reported in prisons worldwide. This study aimed to evaluate the impact of different COVID-19 vaccination strategies in epidemiologically semi-enclosed settings such as prisons, where staff interact regularly with those incarcerated and the wider community.

**Methods:**

We used a metapopulation transmission-dynamic model of a local prison in England and Wales. Two-dose vaccination strategies included no vaccination, vaccination of all individuals who are incarcerated and/or staff, and an age-based approach. Outcomes were quantified in terms of COVID-19-related symptomatic cases, losses in quality-adjusted life-years (QALYs), and deaths.

**Results:**

Compared to no vaccination, vaccinating all people living and working in prison reduced cases, QALY loss and deaths over a one-year period by 41%, 32% and 36% respectively. However, if vaccine introduction was delayed until the start of an outbreak, the impact was negligible.

Vaccinating individuals who are incarcerated and staff over 50 years old averted one death for every 104 vaccination courses administered. All-staff-only strategies reduced cases by up to 5%. Increasing coverage from 30 to 90% among those who are incarcerated reduced cases by around 30 percentage points.

**Conclusions:**

The impact of vaccination in prison settings was highly dependent on early and rapid vaccine delivery. If administered to both those living and working in prison prior to an outbreak occurring, vaccines could substantially reduce COVID-19-related morbidity and mortality in prison settings.

**Supplementary Information:**

The online version contains supplementary material available at 10.1186/s12889-022-13219-4.

## Background

Due to concerns about outbreaks of severe acute respiratory syndrome coronavirus 2 (SARS-CoV-2), many countries introduced non-pharmaceutical interventions (NPIs) in prisons at an early stage of the coronavirus disease 2019 (COVID-19) pandemic. High population turnover, including rooms with multiple occupancy, poor ventilation and poor sanitation can lead to rapid transmission of respiratory pathogens in prison settings [1, 2]. People who are incarcerated are also at greater risk of severe outcomes due to a higher prevalence of underlying health conditions than the general population [3, 4]: it is commonly stated that the average person in prison has the health needs of a person 10 years older in the community [5–7].

In the UK, a range of measures were introduced in prisons from March 2020: all social visits, education, training and employment activities were halted; a “compartmentalisation” strategy was introduced, which involved isolation of symptomatic individuals, shielding of vulnerable individuals and quarantining of people newly transferred to the prison; physical distancing of 2 m amongst both staff and individuals who are incarcerated was also implemented where possible [8]. These measures were supplemented with routine testing of staff and reception testing of those incarcerated from autumn 2020 [9].

Despite these interventions, 19,066 people in custody tested positive for COVID-19 across 128 Prison or Youth Custody Service establishments in England and Wales between 16 March 2020 and 30 September 2021 [10]. A study in the UK has reported 3.33 times higher death rates in UK prisons between March 2020 and Feb 2021 compared to the general population, after adjusting for the age and sex structure of the prison population [11]. Similarly, a study of people hospitalised with COVID-19 in the US found that living in prison was associated with a 2.32 times higher odds of in-hospital death, even after adjusting for age, sex, race and comorbidities [12].

There have also been concerns raised about the impact of the restrictions on the mental health of those living in prison [13].

Due to the elevated risk of transmission and the difficulties of implementing physical distancing measures effectively, the World Health Organization’s Strategic Advisory Group of Experts on Immunisation (WHO-SAGE-I) listed those living and working in detention facilities among the groups that should be prioritised for COVID-19 vaccination [14]. Vaccination could provide a favourable approach to reducing the health burden associated with COVID-19 in prisons, whilst allowing for the relaxation of restrictions and resumption of visits, education and training. The prioritisation of those in prison settings was considered by the Joint Committee on Vaccination and Immunisation (JCVI) in the UK in early 2021. However, it was decided that those living or working in prisons should be vaccinated by age and risk group, in line with the general population [15].

There have been a number of modelling studies investigating SARS-CoV-2 transmission in prisons and detention facilities internationally [16–18], one of which also assesses the impact of vaccination in combination with other interventions [18]. However, no studies have yet been published looking at the impact of vaccination in prisons in England and Wales. In this study, we used a metapopulation model to evaluate the impact of different COVID-19 vaccination strategies in an average local prison in England and Wales, including vaccinating individuals who are incarcerated and/or staff or taking an age-based approach**.** We also looked at how this impact varies with timing of vaccination, to provide insights for future booster vaccination campaigns and outbreak response.

## Methods

### Epidemiological model

We used CovidM, a transmission-dynamic mathematical model for SARS-CoV-2 transmission [19] which has previously been used to evaluate the impact of vaccination in the UK at the population-level [19–21]. It is an SEIR (susceptible, exposed, infectious, recovered) compartmental model with three infectious states to allow for differing levels of infectiousness in people with clinical and subclinical infection. After effective contact with an infectious individual, a susceptible person moves into the exposed state. A proportion of these individuals will move into the preclinical state (Ip), progressing to clinical (Ic). Those in the pre-clinical state are 63% as infectious as those who have already developed symptoms [22]. The remaining exposed individuals will experience subclinical infection (Is) and are assumed to be only 35% as infectious as their clinically-infected counterparts [22]. Individuals with either clinical or subclinical infection then progress to the recovered state, where they are immune to reinfection. Vaccinated individuals move into separate compartments, where they are immune to infection. Both natural and vaccine-induced immunity are assumed to wane exponentially over time, and individuals move from the recovered and vaccinated compartments back into the susceptible compartment.

To account for the semi-enclosed nature of prison settings (from an epidemiological perspective), the prison population was considered as a metapopulation with three interacting sub-populations: (A) Staff Group 1 – staff who have no contact with individuals who are incarcerated; (B) Staff Group 2 – staff who have contact with both (A) Staff Group 1 and those who are incarcerated; and (C) individuals who are incarcerated. Mixing within these three sub-populations was assumed to be homogenous. Contact patterns between the three sub-populations were informed by knowledge of the author working within HMPPS (Table 1). However, further scenarios were considered in the sensitivity analysis: (1) homogenous mixing between staff groups, with no contact between those incarcerated and staff; (2) no contact between Staff Group 1 and the other two subgroups, with homogenous mixing between Staff Group 2 and those who are incarcerated.

**Table 1:** Model parameters, including distributions used in the probabilistic sensitivity analysis

Parameter	Mean (SE)	Distribution	Source and notes
R0	5.08 (1.41)	log-normal	[23]
Percentage reduction in R0 due to shielding and cohorting	40%	beta	[17]
Vaccine efficacy against infection – first dose	60% (10–70%)	beta	[24, 25]
Vaccine efficacy against infection – second dose	80% (10–90%)	beta	
Vaccine efficacy against disease – first dose	60% (50–90%)	beta	
Vaccine efficacy against disease – second dose	85% (50–95%)	beta	
Waning of vaccine immunity	Vaccine efficacy falls by 19% (95%CI 8%-34%) over six months	beta	[26]
Duration of natural immunity	16% (95%CI 13–19%) reduction in immunity over one year	beta	[27]
Staff turnover	8.4% per year (0.1%)	beta	[28]. In-migration assumed to be equal to out-migration
New people incarcerated	0.60% per day (0.17%)	beta	[29]. Based on new receptions into local male prisons. In-migration assumed to be equal to out-migration
Number vaccinated per day	20	fixed	Assumption based on insights of author from HMPPS (O’Mara)
Community incidence	0.001 per 10,000 people per day (0.0015)	fixed	[30]
			[31]
Population size of those incarcerated	820	fixed	[29]. HMPPS Prison Population Tool. Mean for local male prisons
Staff population size – Staff Group 1	70	fixed	[28]. Mean for local male prisons
Staff population size – Staff Group 2	315	fixed	[28]. Mean for local male prisons
Age distribution of people who are incarcerated		fixed	[29]. HMPPS Prison Population Data Tool, December 2020. Mean for local male prisons
Age distribution of staff		fixed	[32]
Contact patterns—Staff Group 1	80% of contacts with other staff in Group 1; 20% with Staff Group 2	fixed	Internal HMPPS correspondence
Contact patterns—Staff Group 2	20% of contacts with Staff Group 1; 40% with other staff in Group 2; 40% with people who are incarcerated	fixed	Internal HMPPS correspondence
Contact patterns—individuals who are incarcerated	40% of contacts with Staff Group 1; 60% of contacts with other people who are incarcerated	fixed	Internal HMPPS correspondence
Quality-Adjusted Life Year (QALY) loss per symptomatic case	0.008 (4.7 × 10–5)	beta	[33]
QALY loss per non-fatal hospitalisation	0.018 (0.0018)	beta	[34]
QALY loss per non-fatal ICU admission	0.154 (0.0304)	beta	[35]
QALY loss per fatality—staff	Age-dependent (SMR = 2, qCM = 0.9, discount rate = 0.35)		[36]
QALY loss per fatality – people who are incarcerated	Age-dependent (SMR = 2.3, qCM = 0.9, discount rate = 0.35)		[36, 37]
QALY loss per Adverse Event Following Immunisation (AEFI)—minor	1/365.25	fixed	[20]
Frequency of AEFI—minor	Age-dependent	fixed	[38]
QALY loss per AEFI—fatal	Age-dependent (SMR = 2, qCM = 0.9, discount rate = 0.35)	fixed	[36]
Frequency of AEFI—fatal	0.18 × 3/1000000	fixed	[39, 40]
Lateral Flow Device (LFD) sensitivity	0.8 (0.125)	beta	[41]
LFD uptake among prison staff	0.508 (0.107)	beta	[9]
Vaccine coverage	0.675 (0.148)	beta	[42]

The prison-specific parameters were chosen to reflect an average local male prison in England and Wales. Staff turnover was estimated using the mean proportion of staff who left their job over one year [28]. Resident turnover was estimated using the mean new reception rate for male local prisons between September-December 2020 [29]. In-migration of new people who are incarcerated and staff was assumed to be equal to out-migration. The population sizes and age distribution for the sub-population who are incarcerated was based on the mean across local male prisons on 31st December 2020 [29]. All three sub-populations were split into five-year age-bands, from ages 15–19 years up to 75 years and older. The population sizes for Staff Group 1 and Staff Group 2 were similarly based on the mean number of staff in post in male local prisons on 31 December 2020, whilst the age distribution was based on mean proportion of people in Staff Group 1 and people in Staff Group 2 per age group in 2019/2020 [32].

### Outbreak timing

Given that the timing of outbreaks varies by prison, the timeline was kept independent from the situation in the wider community in the UK. Community incidence and proportion of the population with prior immunity were varied in the sensitivity analysis. Basic reproduction number (R0) and vaccine efficacy were also varied in order to capture the rapidly changing variant and vaccine landscape.

We allowed introduction of new infections into the prison population via daily contact between staff and the community. The rate of new introductions was based on estimated incidence rate per 10,000 people per day as reported in the Office for National Statistics COVID-19 Infection Survey [30]: mean incidence between June 2020 and February 2022 was used in the base case analysis, but this was varied in the sensitivity analysis. It was assumed that 25% of cases would be detected through regular testing, based on 50.8% uptake of staff Lateral Flow Device (LFD) testing (reported in March 2021) [9], 80% sensitivity for detecting infectious individuals [41] and assuming that those who tested positive would stay home from work.

The basic reproduction number was assumed to have a value of 5 based on a mean estimate for R0 of the delta variant [23], which was varied between 3.2 and 8 in the sensitivity analysis. A scenario in which R0 was reduced by 40% was also considered, to account for the impact of shielding and cohorting [17].

In the base case, the prison population was fully susceptible at t = 0. A scenario analysis was undertaken in which 10%, 30% and 50% of the population were assumed to have prior immunity.

### Vaccination scenarios

Seven COVID-19 vaccination scenarios were considered: no vaccination (1); vaccination of Staff Group 1 only (2); Staff Group 2 only (3); all staff (4); all people who are incarcerated (5); all people living or working in prison who are over 50 years old—reflecting the prioritisation strategy used in initial COVID-19 vaccination rollout in the UK (6) [43]; and all those living or working in prison (7). Vaccination was carried out in campaign mode, assuming a vaccination rate of 20 individuals per day and continuing until uptake of 68% [42] was achieved for all groups vaccinated under that scenario.

In the base case, vaccination was administered prior to an outbreak. A secondary analysis explored the impact of using vaccination as part of outbreak response. Increasing the number of individuals vaccinated per day to 50 was also explored under this scenario.

Coverage among new staff and people who are newly incarcerated was assumed to be the same as coverage achieved inside the prison. Uptake was varied between 36% (median hepatitis B vaccine uptake in prison in England and Wales [44]) and 87% (coverage with at least one dose in people 12 years and older in the UK population as of 2 November 2021 [45]) in the sensitivity analysis. Varying coverage amongst staff and those who are incarcerated independently was also explored.

The base case values used for vaccine effectiveness against infection and disease were based on efficacy against Delta, to ensure consistency with assumptions made about transmissibility and severity [24]. Efficacies were varied across a wide range in the sensitivity analysis to ensure the results remain relevant against the rapidly changing landscape of both vaccines and variants. The ranges chosen are based on efficacies reported against a number of different variants, including Omicron [46, 47]. We assumed a 28-day delay post-immunisation in which vaccine effectiveness remained at 0% [48] and a 14-day delay after the second dose, with a 12-week gap between doses. Vaccine immunity is assumed to fall by 19 percentage points over six months (varied between 8 and 34% in the sensitivity analysis) [26]. Natural immunity was assumed to fall by 15% over one year (varied between 13 and 19% in the sensitivity analysis) [27].

### Outcomes

The impact of each vaccination strategy on cases and deaths was tracked over a one-year period. A longer time horizon of five years was explored in the sensitivity analysis. The health burden was also quantified in terms of quality-adjusted life years (QALYs) lost, taking into account the impact of symptomatic cases, non-fatal hospitalisations, non-fatal admissions to intensive care and adverse events following immunisation (AEFI) [20]. A spreadsheet tool developed by Briggs et al. was used to estimate discounted QALYs associated with premature deaths due to COVID-19 [36]. This approach is further discussed in the supplementary material.

To account for the higher health burden experienced by those who are incarcerated in comparison with the general population, a secondary analysis was run in which the age-specific SARS-CoV-2 infection-fatality ratios were shifted downwards by 10 years [3, 6, 7]. This meant that, for example, people in prison who are aged 50–54 experienced the infection-fatality ratio of those aged 60–64 in the general population.

A probabilistic sensitivity analysis was carried out using Latin hypercube sampling with 500 iterations, with parameter values taken from the distributions described in Table 1. The overall uncertainty in incidence over time was estimated for each vaccination scenario.

Partial rank correlation coefficients (PRCC) were calculated to assess the influence of different parameters on the total cases averted and total QALY loss averted over one year. Univariate sensitivity analyses were then performed for the parameters identified as the most important drivers of uncertainty, to investigate whether variation in these parameters impacted the conclusions made about which vaccination strategies were the most effective.

## Results

**Fig. 1:**
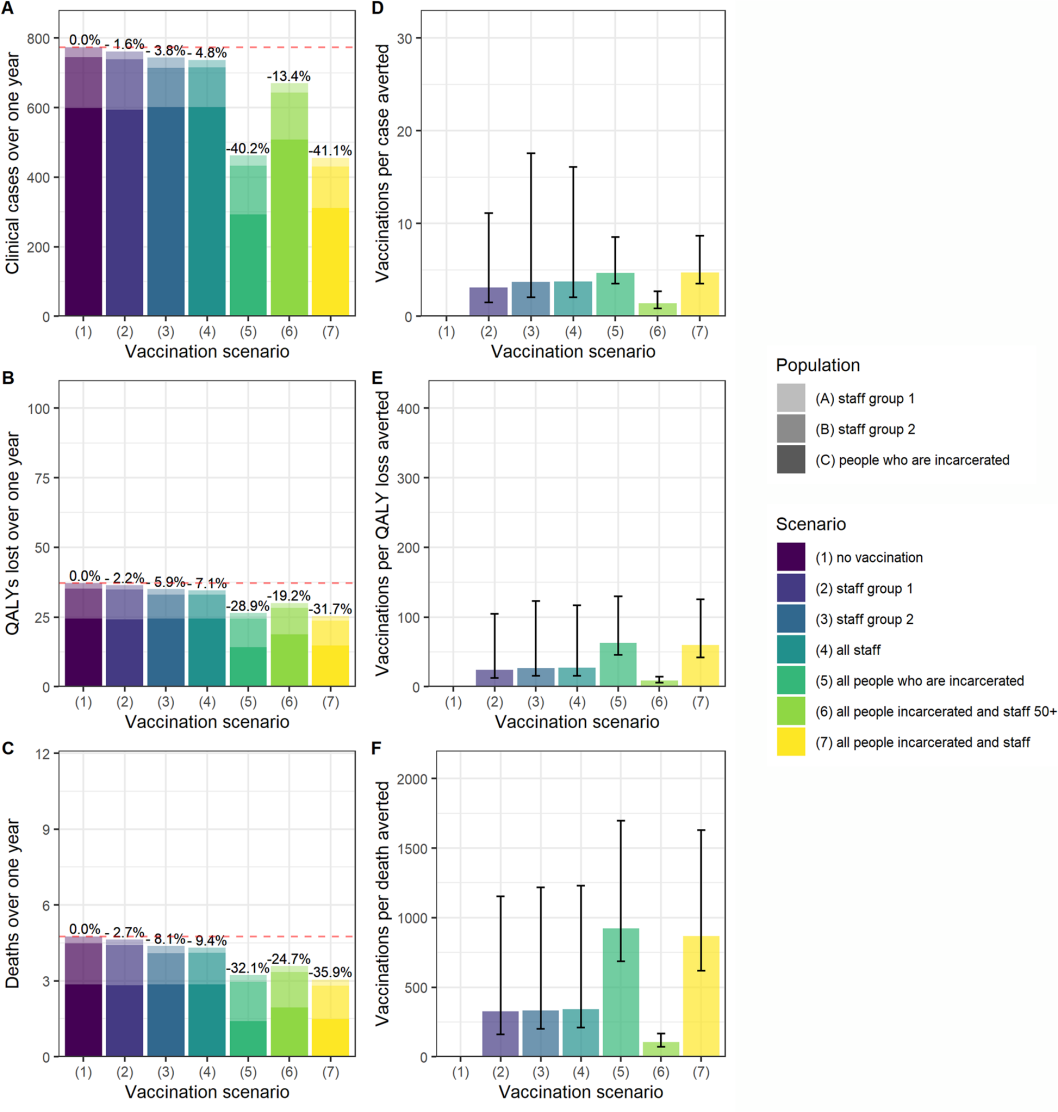
A-C Cases, QALY loss and deaths over one year in an average local male prison, under each vaccination scenario per sub-population and in total. Staff Group 1 are prison staff without contact with those are incarcerated. Staff Group 2 are staff with contact with those who are incarcerated. D-F Vaccination course per case, QALY lost, and death averted over one year under each vaccination scenario. The axes match those used in Fig. 4 for ease of comparison

**Fig. 2:**
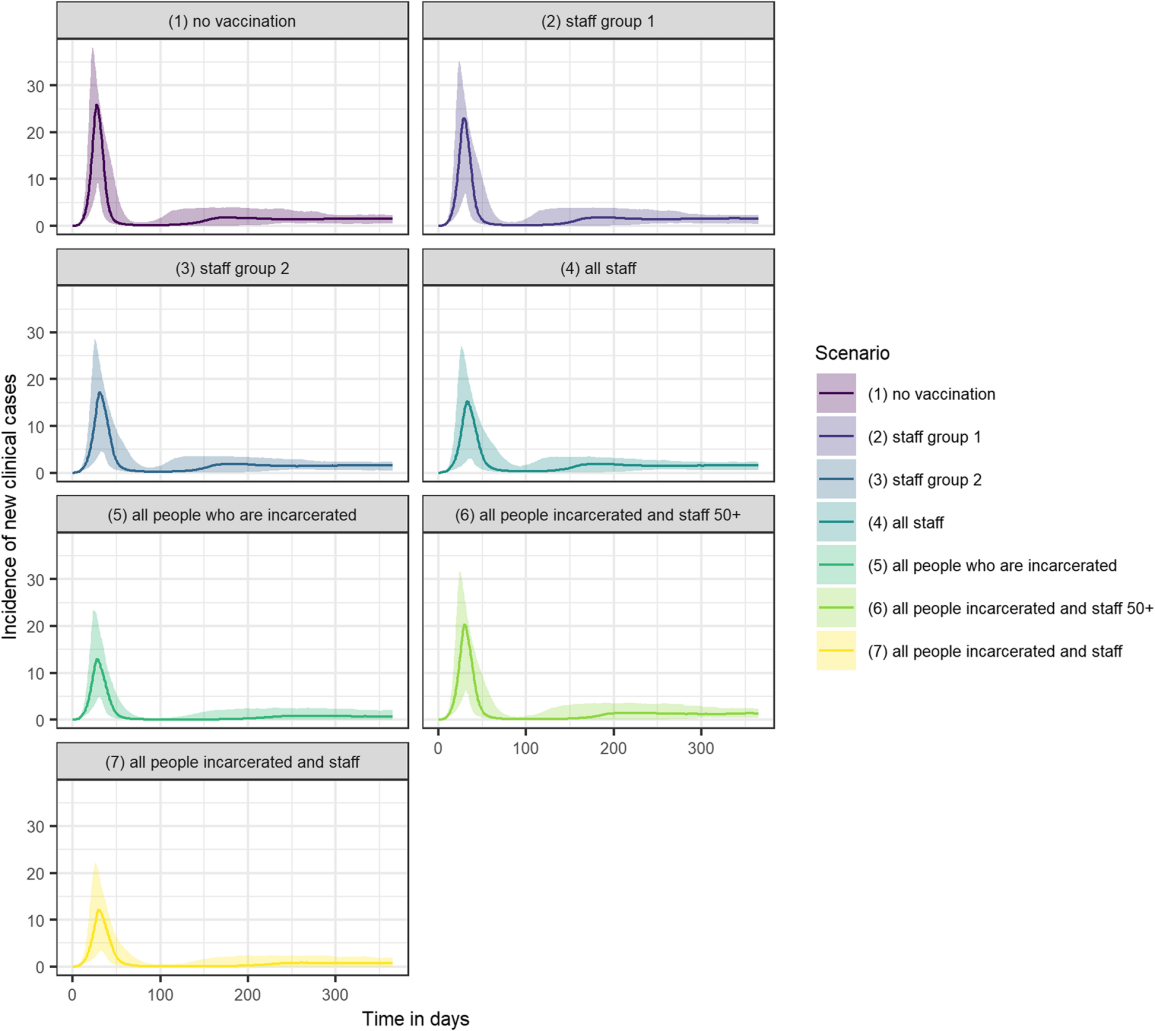
Incidence of new clinical cases over time under each vaccination scenario, including uncertainty captured using probabilistic sensitivity analysis

Vaccinating all people living and working in prison settings reduced cases, QALY loss and deaths by 41.1%, 31.7% and 35.9% respectively, and was the strategy with the largest impact on all three outcomes (Fig. 1A-C). When assuming that R0 = 5, vaccinating all those living and working in prison reduced peak incidence by over half, from 26 new clinical cases per day to 12 (Fig. 2). Only vaccinating individuals who are incarcerated had an almost comparable impact, reducing cases, QALY loss and deaths by 40.2%, 28.9% and 32.1% respectively.

In the base case analysis, staff-only vaccination strategies had a lower impact on cases, QALY loss and deaths than the strategies involving vaccination of people who are incarcerated. Vaccinating all staff reduced overall cases, QALY loss and deaths by 3.8%, 5.9% and 8.1% (Fig. 1A-C).

In terms of the vaccination courses administered per case, QALY loss and death averted, vaccinating all those living and working in prison over 50 years old was the most efficient strategy (Fig. 1D-F). Under this scenario, one death was averted for every 104 (95%UI 71–167) courses of vaccination administered (Fig. 1F).

### Scenario analysis

**Fig. 3:**
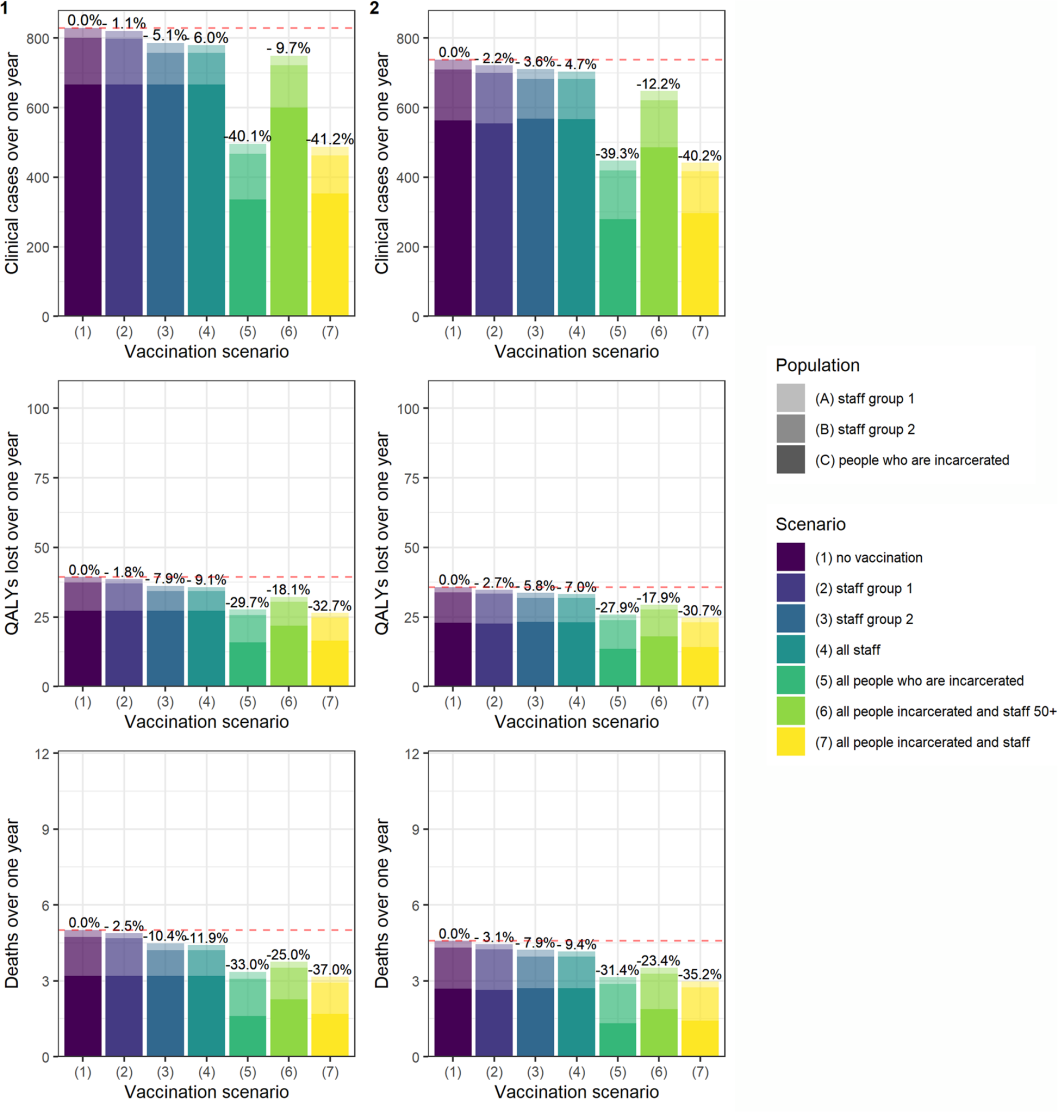
Cases, QALY loss and deaths over one year under differing assumptions about mixing between sub-groups. Scenario (1): homogenous mixing amongst staff; no contact with those who are incarcerated. Scenario (2): homogenous mixing amongst Staff Group 2 and those who are incarcerated; no contact with Staff Group 1. The axes match those of Fig. 4 for ease of comparison

The findings were robust to variation in mixing between sub-groups: strategies involving individuals who are incarcerated remained more effective under both scenario 1 (homogenous mixing among staff with no contact with those who are incarcerated) and scenario 2 (homogenous mixing among Staff Group 2 and those who are incarcerated with no contact with Staff Group 1) (Fig. 3).

**Fig. 4:**
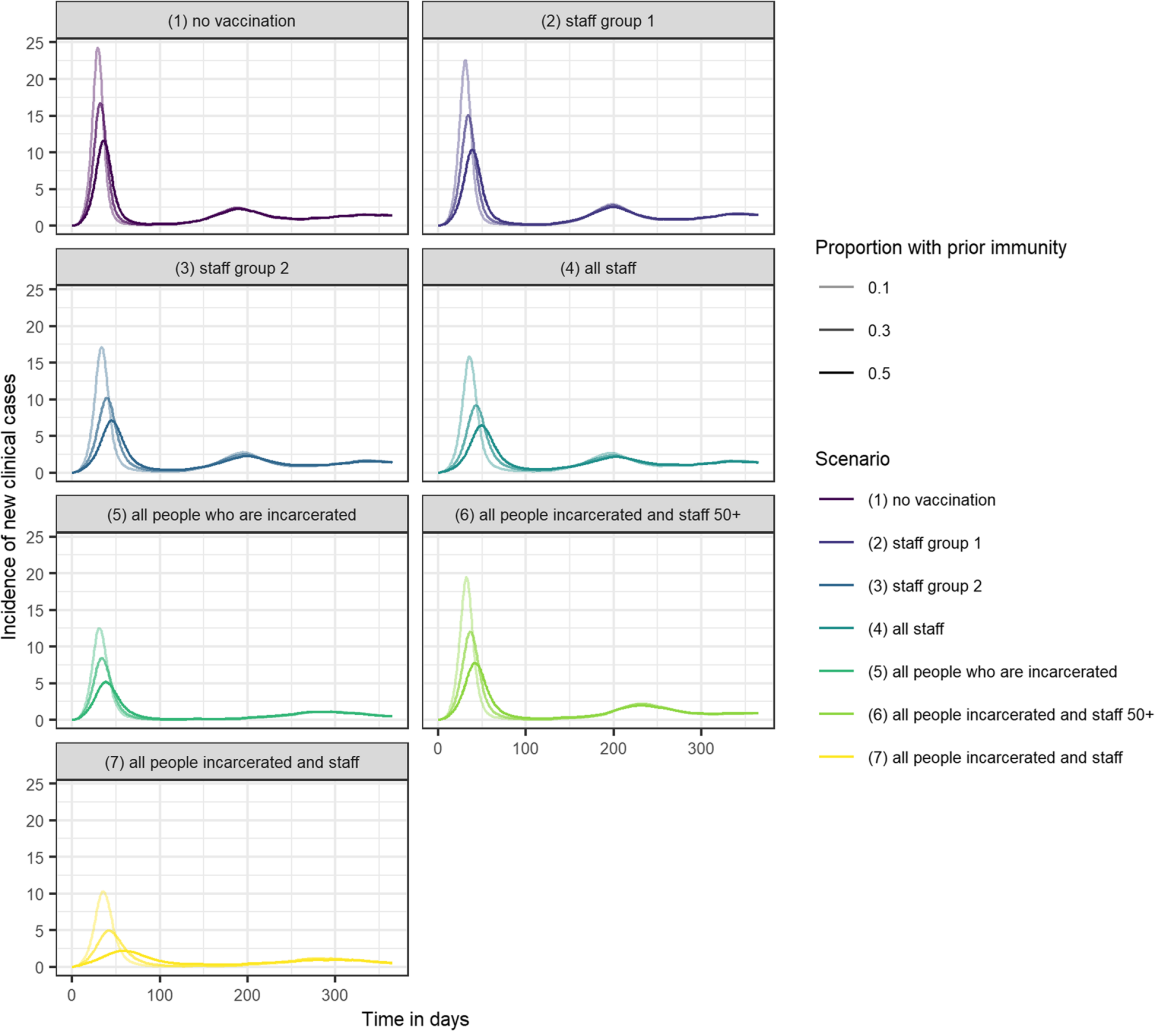
Incidence of new clinical cases over one year in an average local male prison, under seven different vaccination scenarios. Assuming 10%, 30% and 50% of the population have prior natural immunity

Greater infection-induced immunity prior to the outbreak also reduced the impact of vaccination strategies, but the relative impact of scenario 7 compared to all other strategies was increased. Assuming 50% prior immunity, scenario 5 reduced peak incidence to five cases per day, whereas scenario 7 reduced peak incidence to 2.5 cases per day (Fig. 4).

**Fig. 5:**
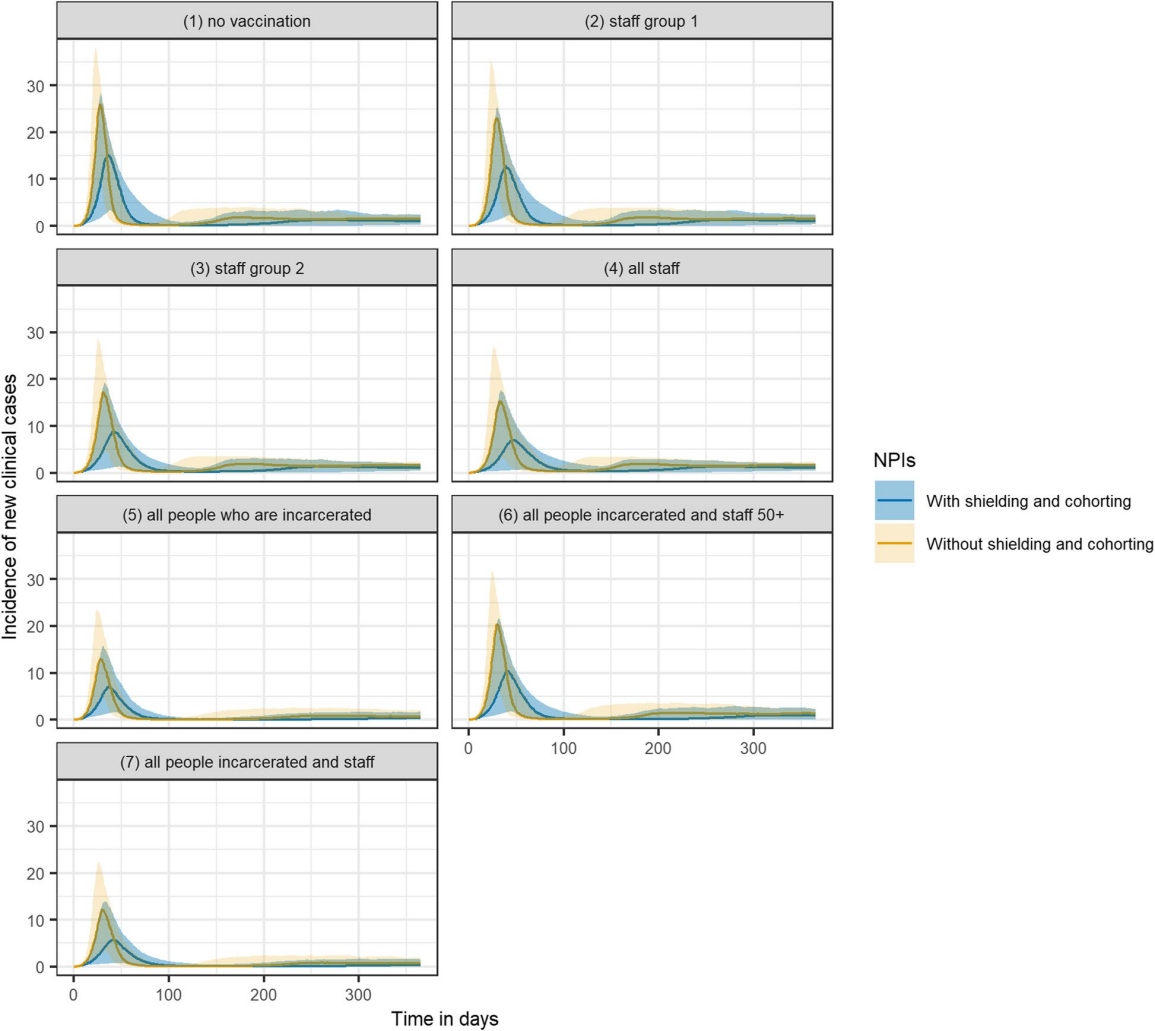
Incidence of new clinical cases over time under each vaccination scenario and under different values of R0

At lower values of R0 (i.e., when vaccination was combined with shielding and cohorting) the difference in peak incidence between scenarios was reduced (Fig. 5). However, combining vaccination of all people living or working in prison with these non-pharmaceutical interventions meant that peak incidence was five new clinical cases per day, compared to 12 cases and 15 cases when using vaccination or NPIs independently.

When vaccination was introduced in response to an outbreak rather than prior to introduction of infection, peak incidence remained at around 26 new clinical cases per day under all scenarios (Supplementary Fig. 1, Additional File 1), even if vaccination rate was increased to 50 per day (Supplementary Fig. 2, Additional File 1).

**Fig. 6:**
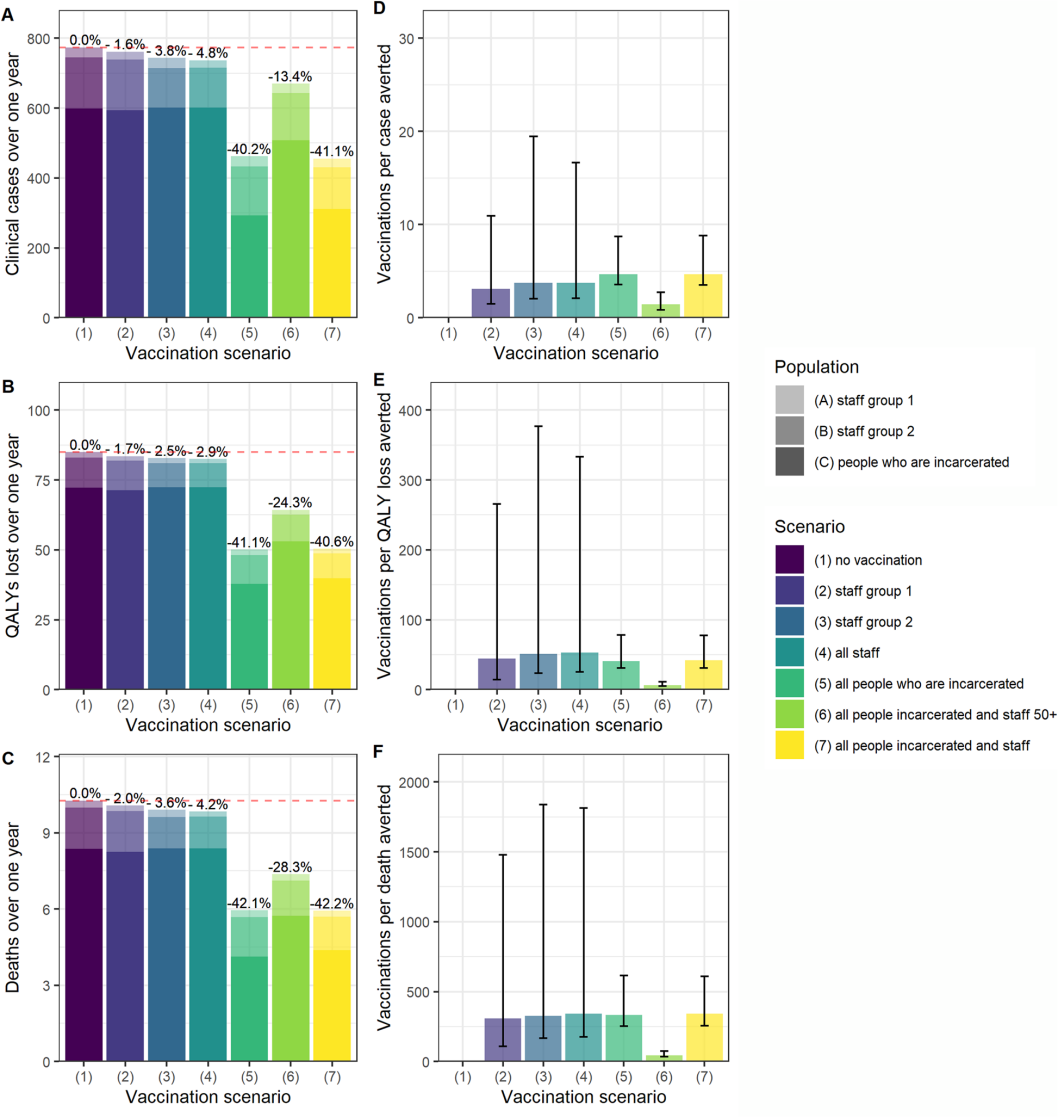
**A**-**C** Cases, QALY loss and deaths over one year under each vaccination scenario, when assuming a higher IFR in those who are incarcerated. **D-F** Vaccination courses per case, QALY loss and death averted, when assuming higher IFR in those who are incarcerated

When people who are incarcerated were assumed to have IFRs in line with those who are 10 years older in the general population, there was an increase in QALYs lost and deaths over one year. The percentage reduction in QALYs lost and deaths increased in strategies involving vaccination of people who are incarcerated (Fig. 6). The efficiency of these strategies also increased: one death was averted for every 45 (95%UI 34–74) people vaccinated under scenario 6, compared to 104 (95%CI 71–167) in the base case analysis.

### Probabilistic sensitivity analysis

**Fig. 7:**
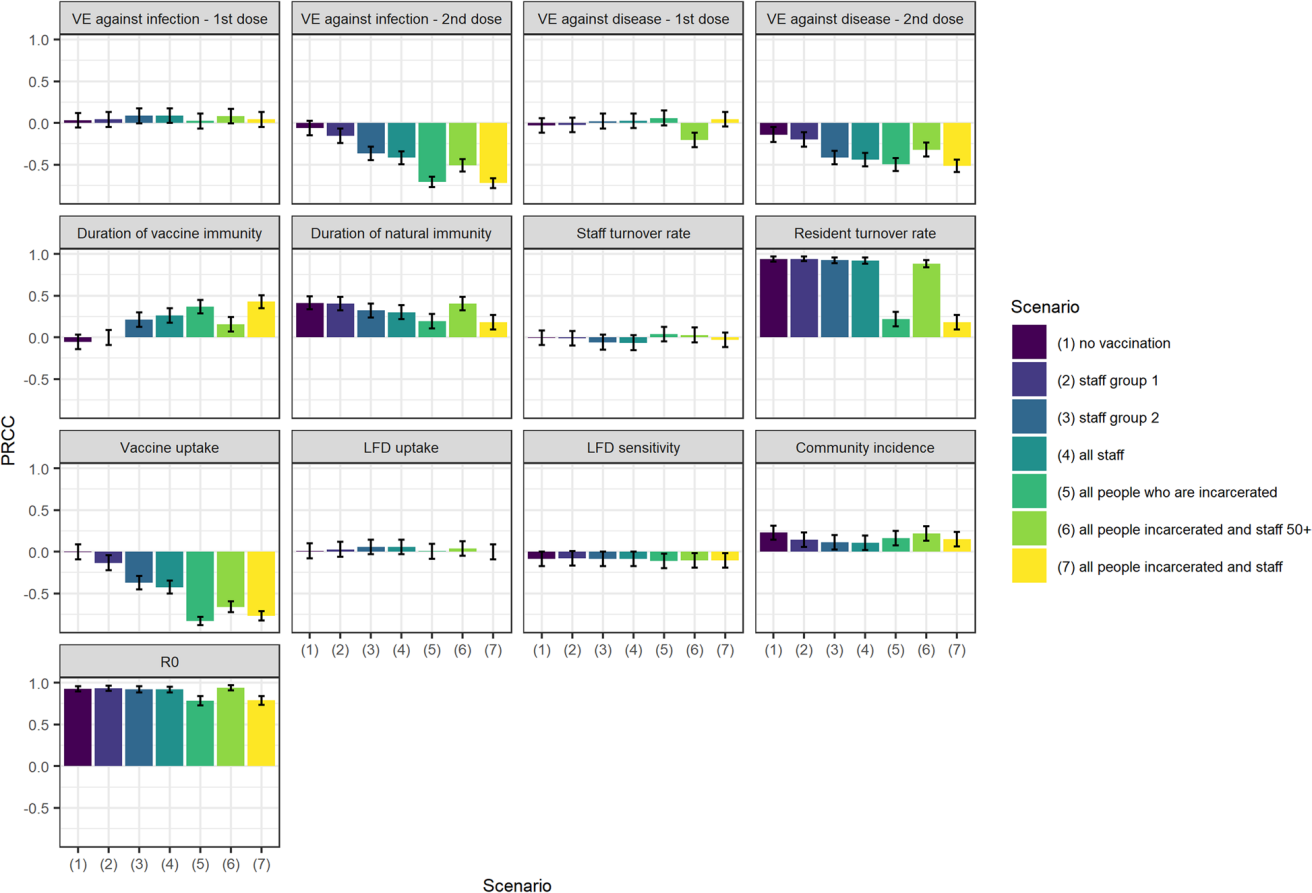
Sensitivity of estimated total cases averted under each vaccination scenario to variation in parameters. Bars show PRCCs corresponding to each parameter varied in probabilistic sensitivity analysis

The PRCC indices suggest that the most important drivers of uncertainty in the estimate for total cases averted over one year are resident turnover rate, R0, duration of natural and vaccine immunity, vaccine uptake and vaccine efficacy against disease (Fig. 7). These parameters also had the most influence on QALY loss averted (Supplementary Fig. 4, Additional File 1).

The conclusions in terms of which strategies are the most effective on the whole remain robust to variation in resident turnover rate, duration of vaccine and natural immunity and vaccine efficacy against disease (Supplementary Fig. 5, Additional File 1). However, the difference in cases averted between scenarios is reduced at lower resident turnover, lower duration of vaccine immunity and higher duration of natural immunity (Fig. 6).

Resident turnover becomes less important under scenarios that involve vaccination of those who are incarcerated. As would be expected, uncertainty in duration of vaccine immunity, efficacy against disease and vaccine uptake have greater influence under the scenarios that involve vaccination of those who are incarcerated, as this sub-group constitutes a larger proportion of the overall prison population (Fig. 7). Independently varying uptake amongst staff and those who are incarcerated also illustrates this. Increasing coverage in staff has a negligible impact on overall health burden. By contrast, under scenario 7, increasing coverage amongst those who are incarcerated from 30 to 90% is associated with reduction in cases of around 30 percentage points (Supplementary Fig. 3, Additional File 1).

When the time horizon was increased to five years, waning of natural and vaccine immunity had a large impact on the number of clinical cases over five years, but not on the relative effectiveness of different vaccination scenarios (Supplementary Fig. 7, Additional File 1).

## Discussion

This study explored the impact of seven different COVID-19 vaccination prioritisation strategies in prisons in England and Wales. Our findings show that vaccinating all those living or working in prison could reduce cases and deaths by over 40% and halve peak incidence. If extrapolated across all 32 local prisons in England and Wales [49], this would correspond to over 10,000 cases and 50 deaths averted and 375 QALYs gained over one year, compared to a counterfactual in which shielding and cohorting are not used.

However, the results were sensitive to the timing of vaccine delivery. If vaccination occurred after an outbreak of the current circulating variant had occurred, the impact was negligible. The impact achieved was also strongly dependent on coverage among those who are incarcerated: strategies involving staff only had a much lower impact on cases, QALYs and deaths. Both of these findings—the importance of timely vaccination to avoid large outbreaks and the substantially greater impact achieved by increasing coverage in the prison population—could have implications for the rollout of future booster vaccination campaigns or new variant-specific vaccines.

In terms of the largest impact per vaccination course administered, the most efficient strategy was vaccination of all those living or working in prison who are over 50 years old. Therefore this could be a valuable strategy in settings where supply is constrained.

### Interpretation

In the UK, the Joint Committee on Vaccination and Immunisation (JCVI) advised that those living or working in prison should be vaccinated along with their age and risk group, in line with the general population. The results of this study suggest that vaccinating both those living or working in prison according to their age could have had more impact on morbidity and mortality than any strategy targeting staff only, but suggests that greater impact could have been achieved by rapidly increasing coverage among individuals who are incarcerated.

As illustrated by the high COVID-19 case and death rates in UK prisons despite physical distancing measures, effectively reducing R0 through non-pharmaceutical interventions can be challenging in prison settings [11]. Moreover, implementation of strict non-pharmaceutical interventions in prison settings can have implications for the physical and mental health of those who are incarcerated [13]. Following prison visits in March 2020, the Prison Reform Trust reported that the majority of those incarcerated were restricted to their cells for over 23 h a day [50]. Long-term solitary confinement has been associated with increased risk of mental illness, such as clinical depression and impulse-control disorder, even in people with no prior history of psychiatric problems [51]. The suspension of visits from family members and educational activities could also impact the prevalence of depression and rates of re-offending [13, 52]. Therefore, increasing vaccination coverage in those who are incarcerated may not just avert morbidity and mortality associated with COVID-19, but reduce reliance on restrictions that have a negative impact on mental health.

### Strengths and limitations

The model includes staff and allows for importation of cases via contact between staff and the community. Observational studies in the US have identified staff as an important source of introductions of SARS-CoV-2 infections into prisons and detention facilities [53]. The model does not account for risk of SARS-CoV-2 importation via new residents, as a previous study found that the reverse cohorting strategies used in prisons in England and Wales could detect up to 99% of incoming infections if new arrivals were required to self-isolate for 14 days [17]. This therefore suggests importation of cases via new residents is not an important driver of outbreaks in prisons using these strategies. The assumption that there is no contact between those living in prison and the community may also be less appropriate now that family visits have resumed.

The approach taken to account for the higher prevalence of underlying conditions among those who are incarcerated uses a widely-cited finding that the general health of a 50-year-old resident in prison is similar to that of someone then years their senior in the community [7]. Whilst this is a crude estimate [54], it does provide a way of assessing how worse physical health among those who are incarcerated in comparison to the general population may impact the morbidity and mortality associated with different vaccination strategies. Additionally, there is evidence from studies in the US and UK that rates of ICU admission, in-hospital mortality and 30-day mortality are higher among the prison population [11, 12] suggesting that the elevated risk of severe outcomes among those who are incarcerated is an important factor to consider when determining whether they should be prioritised for vaccination. Other studies have been more specific: Ryckman and colleagues had access to resident-level data on health characteristics [18]; Bays and colleagues used average data provided by HMPPS to estimate that 1.2% of the prison population were extremely clinically vulnerable, but excluded these individuals from the analysis under the assumption that shielding was 100% [17]. However, we did not have access to individual-level data as used by Ryckman and colleagues, and only considering those who are extremely clinically vulnerable may not fully capture the increased risk of severe outcomes within the prison population.

Increased risk of cardiovascular or thromboembolic events following SARS-CoV-2 infection, as well as other longer term symptoms such as fatigue, were not included in the QALY calculations [55]. Therefore the estimated impact of vaccination on QALY loss averted is likely to have been underestimated.

In terms of the health burden associated with AEFIs, only minor systemic and fatal events were considered. However, an estimate of one Quality-Adjusted Life Day lost on average per minor AEFI is likely to be conservative and therefore will go some way to capturing the impact of other AEFIs. The inclusion of more severe AEFIs such as myocarditis or thromboembolism were considered, but given these where not included in the QALY loss calculations associated with SARS-CoV-2 infection, their inclusion as AEFIs would introduce bias.

The scarcity of data on contact patterns between sub-populations in prison was also a key limitation: the mixing patterns used had to be informed by expert opinion rather than empirical estimates. However, varying these contact patterns did not substantially alter the conclusions in terms of the relative effectiveness of each vaccination strategy.

Also due to limited data on contact patterns, the model structure did not account for room occupancy structure. This could be an important limitation, given that previous studies have found a significant difference between infection rates in residents of dormitories (with three or more occupants) versus cells (with up to two) [2]. For the same reason, prison layout was also not considered. This could mean that the size of outbreaks may be overestimated: for example, if there is an outbreak in one wing but insufficient contact with other wings for the outbreak to span the whole prison. However, given the frequency of imported cases via infected staff, it is likely that all wings would be exposed at some point, even if asynchronously.

There also remains substantial uncertainty around the value of the vaccine-related parameters. The large influence of vaccine efficacy against disease (2nd dose) on uncertainty in outcomes is important to note, given that this is a parameter that has previously decreased with the emergence of new variants [24]. However, varying vaccine efficacy and duration of immunity in the sensitivity analyses did not alter the key conclusions about which strategies could be the most effective at reducing total cases. In fact, a number of the parameters that were the greatest drivers of uncertainty were those that could potentially be altered via other interventions: resident turnover, vaccine uptake and R0.

The strong influence of resident turnover on total cases averted could be an important consideration when looking to generalise these findings across different prison types. This analysis only considered local prisons, which serve the courts and have a high turnover. The results therefore may differ for other prison types, such as high security or open prisons.

Vaccine uptake was assumed to be 68% based on uptake in prisons in California [42]. Uptake was also assumed to be the same amongst staff and those who are incarcerated. According to a single datapoint available from October 2021, 60% and 66% of the prison populations in England and Wales had received their first dose respectively, whilst 51% and 56% had received their second dose [56]. Coverage was lower amongst staff: up to 24th September 2021, 50% of staff have received their first dose and 41% had received their second. More up-to-date data is not publicly available and nevertheless, coverage will likely vary on a prison-by-prison basis. The scenario analysis in which uptake in staff and resident populations were varied independently broadly captures all likely possibilities.

The impact of specific NPIs, such as isolation of those who are infected, was not explored explicitly within the model structure. Instead it was assumed introduction of NPIs will lower R0, informed by the reduction in R0 due to NPIs reported in a previous study in UK prisons [17]. The R0 assumed in the base case was also informed by estimates for the Delta variant in the community rather than specifically in prison settings [23]. However, a large range of values was considered in the sensitivity analysis.

An important additional benefit of vaccinating prison populations that was not accounted for in this model was the impact of outbreaks in prisons on transmission in the community. Previous mathematical modelling studies investigating effectiveness and cost-effectiveness of prison-based interventions against a range of diseases have taken into account contact between prison populations and the general population [57]. The impact of transmission from prisons to the community is likely to be an even more important factor as prison visits resume and more staff return to working in prisons - as for much of the pandemic only essential staff have been working onsite. Transmission within the prison population and broader community could also be more closely linked in the case of open prisons, in which people living in prison are more likely  to leave the prison during the day on temporary license.

Finally, the model did not consider the economic costs: from a healthcare perspective, for HMPPS, or indeed for those who are incarcerated if illness or NPIs cause delays in court proceedings. This would be a logical next step for future analyses, whilst considering vaccination in combination with more specific sets of NPIs.

### Context of other research

Whilst we did not benefit from the granularity of data available to Ryckman and colleagues, a number of our key findings concurred with their conclusions. They found that increasing vaccination in staff did not have a substantial impact on infections and hospitalisations among residents, just as we found that staff-only strategies were not found to have a large impact on cases, QALYs and deaths among those who are incarcerated. They also found that vaccination timing was important: outbreaks were estimated to be twice as large if vaccination was carried out during rather than prior to introduction of infection; just as we found that vaccination did not appreciably alter the outbreak trajectory if only implemented once infection was introduced.

This is the first model-based analysis of COVID-19 vaccination strategies in prisons in England and Wales. A previous modelling study has investigated the impact of specific NPIs [17], in which they explicitly included isolation within the model structure. Future studies could combine these two approaches to further investigate the impact of vaccination in combination with specific sets of NPIs.

### Generalisability

Varying vaccine efficacy, duration of vaccine immunity, prior infection-induced immunity and transmissibility of large ranges was intended to increase the generalisability of the findings in the context of new variants or new vaccine formulations.

Other countries and regions have taken differing approaches to vaccination in prisons: some are prioritising the vaccination of people who are incarcerated, others are taking a staff-only approach and others have not specifically included prisons in their vaccination strategy [58]. The size and structure of the prisons, as well as the non-pharmaceutical interventions in place, will vary by country. Assumptions made about the level of community incidence and hospitalisation rates, as well as differences in age structure, may lead to different results internationally. Access to vaccines and prior vaccine coverage among those living and working in prisons also differs substantially.

However, the general findings may still be valuable in other settings, such as the substantially greater benefit of vaccinating prison populations prior to introduction of infection. Globally equitable vaccine access is needed to ensure that vaccines reach people most in need, but the finding that an age-based approach is the most efficient strategy (in terms of cases, QALY loss and deaths averted per vaccination course) may be particularly valuable in countries where supply remains more constrained.

The approach taken in which prisons are considered a metapopulation, with people who are incarcerated and staff groups who have different contact rates with each other and the community, could also be extended to investigate the impact of COVID-19 outbreaks in prisons on community transmission.

## Conclusions

Our findings suggest that any vaccination strategy including both staff and people who are incarcerated is the most effective at reducing cases, health loss (in terms of QALYs), and deaths in prison settings. However, the impact of all strategies was dependent on timely vaccine delivery.

Staff-only strategies had a negligible indirect benefit in terms of reduction in cases amongst those who are incarcerated. Therefore, going forward, increasing vaccination coverage among those who are incarcerated, through increasing uptake in those yet to receive vaccine doses or with future booster campaigns, could have a substantial impact on the COVID-19 burden in prison settings.

Introducing vaccination at the start of an outbreak did not have an appreciable impact on cases regardless of the vaccination strategy taken. Therefore delivery of vaccines in prison settings must take place prior to introduction of infection to prevent large outbreaks; if vaccination is introduced too late, strict NPIs would likely also be required.

For settings where vaccine supply remains constrained, the most efficient strategy was found to be vaccination of all those living or working in prison aged 50 years or older. 

## Supplementary Information

**Additional file 1:** **Supplementary Table 1.** Age distribution of people living and working in an average local male prison**.** **Supplementary Table 2.** Input data on age-specific susceptibility to infection and severity of disease. **Supplementary Table 3.** Additional epidemiological parameters. **Supplementary Figure 1.** Incidence of new clinical cases over time under each vaccination scenario, if vaccination is introduced at the start of an outbreak. **Supplementary Figure 2.** Incidence of new clinical cases over time under each vaccination scenario, if vaccination is introduced at the start of an outbreak and administered at a rate of 50 doses per day. **Supplementary Figure 3.** Independently varying vaccine uptake between 30% and 90% among staff and individuals who are incarcerated. **Supplementary Figure 4.** Sensitivity of estimated total QALY loss averted under each vaccination scenario to variation in parameters. **Supplementary Figure 5.** Change in cases averted over one year with change in resident turnover, efficacy against disease (2nd dose), duration of vaccine immunity and duration of natural immunity**. Supplementary Figure 6.** Sum of clinical cases over five years when vaccine and natural immunity are assumed not to wane vs. waning of natural immunity of 16% over one year and waning of vaccine immunity of 19% over six months. **Supplementary Figure 7.** Incidence of new clinical cases over five years under each vaccination scenario, including uncertainty captured using probabilistic sensitivity analysis. **Supplementary Figure 8.** Cases over one year in an average local male prison, by sub-population, under each of seven vaccination scenarios. **Supplementary Figure 9.** QALY loss over one year in an average local male prison, by sub-population, under each of the seven vaccination scenarios. **Supplementary Figure 10.** Deaths over one year in an average male prison, by sub-population, under each of seven vaccination scenarios.

## Data Availability

The data generated during this study and the code for reproducing the analyses is available at https://github.com/ciaramccarthy1/prisons-vacc-strategies.
