# Supplementary material for: The impact of COVID-19 vaccination in prisons in England and Wales: a metapopulation model
Source: BMC Public Health. 2022 May 18;22:1003. doi: 10.1186/s12889-022-13219-4 (PMC9115545; doi:10.1186/s12889-022-13219-4)
Supplement: Supplementary file 1 — Additional file 1: Supplementary Table 1. Age distribution of people living and working in an average local male prison. Supplementary Table 2. Input data on age-specific susceptibility to infection and severity of disease. Supplementary Table 3. Additional epidemiological parameters. Supplementary Figure 1. Incidence of new clinical cases over time under each vaccination scenario, if vaccination is introduced at the start of an outbreak. Supplementary Figure 2. Incidence of new clinical cases over time under each vaccination scenario, if vaccination is introduced at the start of an outbreak and administered at a rate of 50 doses per day. Supplementary Figure 3. Independently varying vaccine uptake between 30% and 90% among staff and individuals who are incarcerated. Supplementary Figure 4. Sensitivity of estimated total QALY loss averted under each vaccination scenario to variation in parameters. Supplementary Figure 5. Change in cases averted over one year with change in resident turnover, efficacy against disease (2nd dose), duration of vaccine immunity and duration of natural immunity. Supplementary Figure 6. Sum of clinical cases over five years when vaccine and natural immunity are assumed not to wane vs. waning of natural immunity of 16% over one year and waning of vaccine immunity of 19% over six months. Supplementary Figure 7. Incidence of new clinical cases over five years under each vaccination scenario, including uncertainty captured using probabilistic sensitivity analysis. Supplementary Figure 8. Cases over one year in an average local male prison, by sub-population, under each of seven vaccination scenarios. Supplementary Figure 9. QALY loss over one year in an average local male prison, by sub-population, under each of the seven vaccination scenarios. Supplementary Figure 10. Deaths over one year in an average male prison, by sub-population, under each of seven vaccination scenarios. [file 12889_2022_13219_MOESM1_ESM.docx]

**Additional File 1**

Supplementary Table 1. Age distribution of people living and working in an average local male prison.

| Population structure | | |
| --- | --- | --- |
| **Age group** | **People living in prisons** | **Prison staff** |
| 0-4 | 0.0000 | 0.0000 |
| 5-9 | 0.0000 | 0.0000 |
| 10-14 | 0.0000 | 0.0000 |
| 15-19 | 0.0643 | 0.0658 |
| 20-24 | 0.1238 | 0.0658 |
| 25-29 | 0.1869 | 0.0658 |
| 30-34 | 0.1663 | 0.1171 |
| 35-39 | 0.1663 | 0.1171 |
| 40-44 | 0.0892 | 0.1097 |
| 45-49 | 0.0892 | 0.1097 |
| 50-54 | 0.0394 | 0.1354 |
| 55-59 | 0.0394 | 0.1354 |
| 60-64 | 0.0115 | 0.0196 |
| 65-69 | 0.0115 | 0.0196 |
| 70-74 | 0.0061 | 0.0196 |
| 75+ | 0.0061 | 0.0196 |

**Epidemiological input parameters**

The susceptibility to infection upon contact and the probability of clinical symptoms on infection were taken from Davies *et al.* 2020 (1). To estimate the infection-hospitalisation risk and infection-fatality risk associated with the Delta variant, we used the absolute risk of hospital admission for after a positive test and death within 28 days after a positive test (2). To estimate IHR and IFR for those aged 75 and over, the mean of estimates for the 70-79 and 80+ agebands was weighted using 2022 population projections for England and Wales (3).

The proportion of hospitalised cases requiring ICU admission was taken from Docherty *et al.* 2020 (4).

Supplementary Table 2. Input data on age-specific susceptibility to infection and severity of disease.

| **Age group** | **Susceptibility to infection on contact** | **Probability of clinical symptoms given infection** | **IHRs (%)** | **IFRs (%)** |
| --- | --- | --- | --- | --- |
| 0-4 | 0 | 0.2904 | 1.0 | 0.003 |
| 5-9 | 0 | 0.2904 | 1.0 | 0.001 |
| 10-14 | 0 | 0.2070 | 0.7 | 0.001 |
| 15-19 | 0.3815 | 0.2070 | 0.7 | 0.003 |
| 20-24 | 0.7860 | 0.2676 | 1.5 | 0.006 |
| 25-29 | 0.7860 | 0.2676 | 1.5 | 0.013 |
| 30-34 | 0.8586 | 0.3284 | 2.6 | 0.024 |
| 35-39 | 0.8586 | 0.3284 | 2.6 | 0.040 |
| 40-44 | 0.7981 | 0.3979 | 3.4 | 0.075 |
| 45-49 | 0.7981 | 0.3979 | 3.4 | 0.121 |
| 50-54 | 0.8167 | 0.4863 | 4.9 | 0.207 |
| 55-59 | 0.8167 | 0.4863 | 4.9 | 0.323 |
| 60-64 | 0.8785 | 0.6307 | 7.1 | 0.456 |
| 65-69 | 0.8785 | 0.6307 | 7.1 | 1.075 |
| 70-74 | 0.7383 | 0.6907 | 12.5 | 1.674 |
| 75+ | 0.7383 | 0.6907 | 14.6 | 11.64 |

Supplementary Table 3. Additional epidemiological parameters

| **Parameter** | **Value** | **Distribution** | **Source** |
| --- | --- | --- | --- |
| Latent period (E to Ip and E to Is; days) | 4.0 | gamma | Davies et al. 2020 |
| Duration of preclinical infectiousness (Ip to Ic; days) | 1.5 | gamma | Davies et al. 2020 |
| Duration of clinical infectiousness (Ic to R; days) | 3.5 | gamma | Davies et al. 2020 |
| Duration of subclinical infectiousness (Is to R; days) | 5.0 | gamma | Davies et al. 2020 |
| Delay from onset to hospitalisation (days) | 7.0 | gamma | Davies et al. 2020 |
| Duration of hospitalisation in ICU bed (days) | 10.0 | gamma | Davies et al. 2020 |
| Proportion of cases that require ICU admission | 0.17 | fixed | Docherty *et al.* 2020 |
| Delay from onset to death (days) | 22.0 | gamma | Davies et al. 2020 |

**Discounting QALYs**

The spreadsheet tool developed by Briggs et al. builds on standard life table methods and adjusts for the impact of comorbidities on mortality and quality of life (5). A standardised mortality ratio (SMR) term is applied to the underlying death rate: here assumed to be 2.3 for those who are incarcerated based on the SMR reported for male prisons in Scotland (6). An SMR of 2 was assumed for the staff population.

Quality-adjusted life expectancy was adapted to account for assumed higher prevalence of comorbidities among those who have died from COVID-19, using a parameter qCM, which refers to the health-related quality of life for a person with a comorbidity associated with increased risk of COVID-19 compared to a person without (5). For example, it has been estimated that those with asthma, heart disease and hypertension have 88%, 82% and 93% of the quality of life of those without these conditions (7). qCM was assumed to be 90%, as a conservative estimate for the reduced quality of life of people at increased risk of COVID-19.


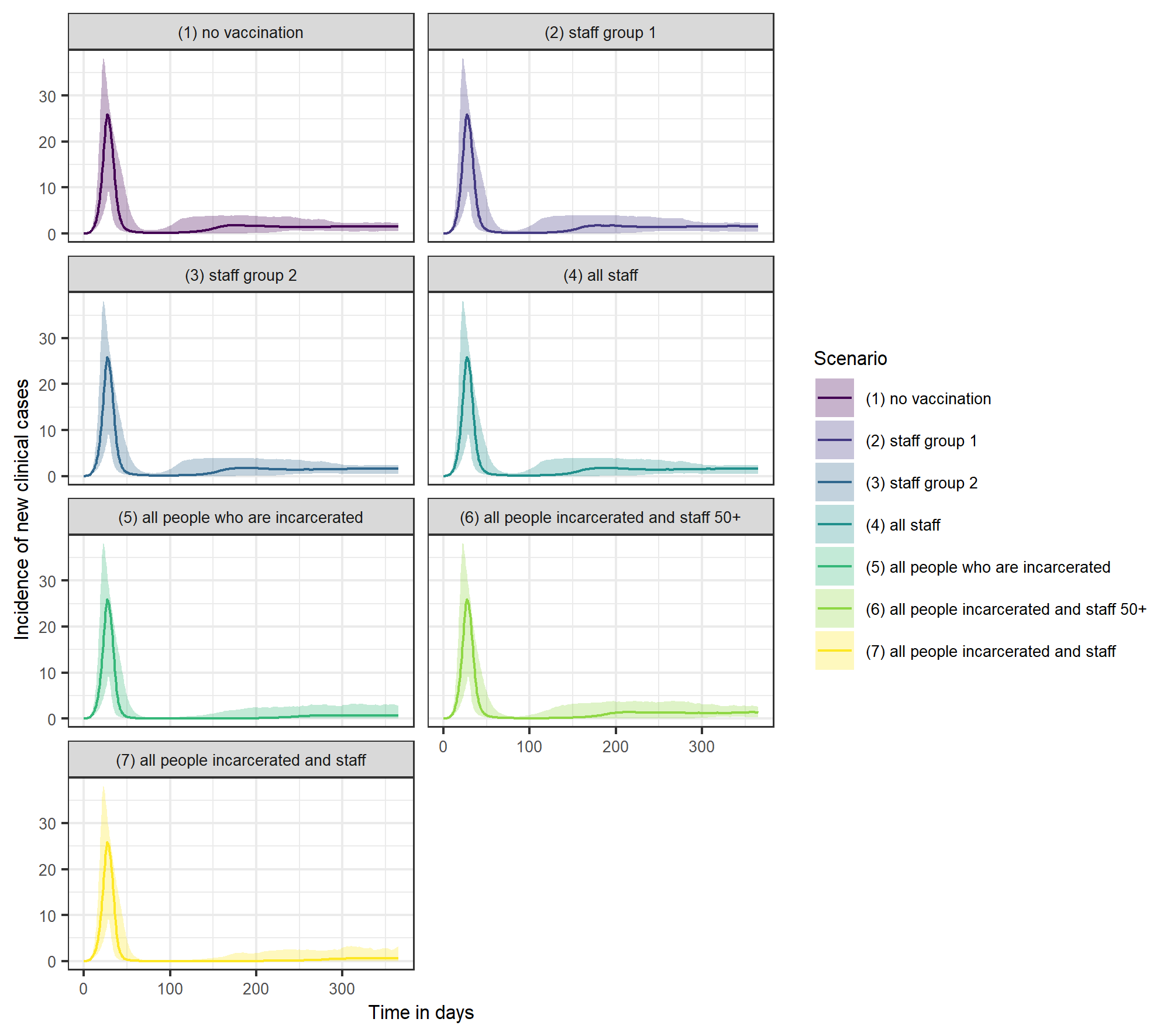


Supplementary Figure 1. Incidence of new clinical cases over time under each vaccination scenario, if vaccination is introduced at the start of an outbreak.


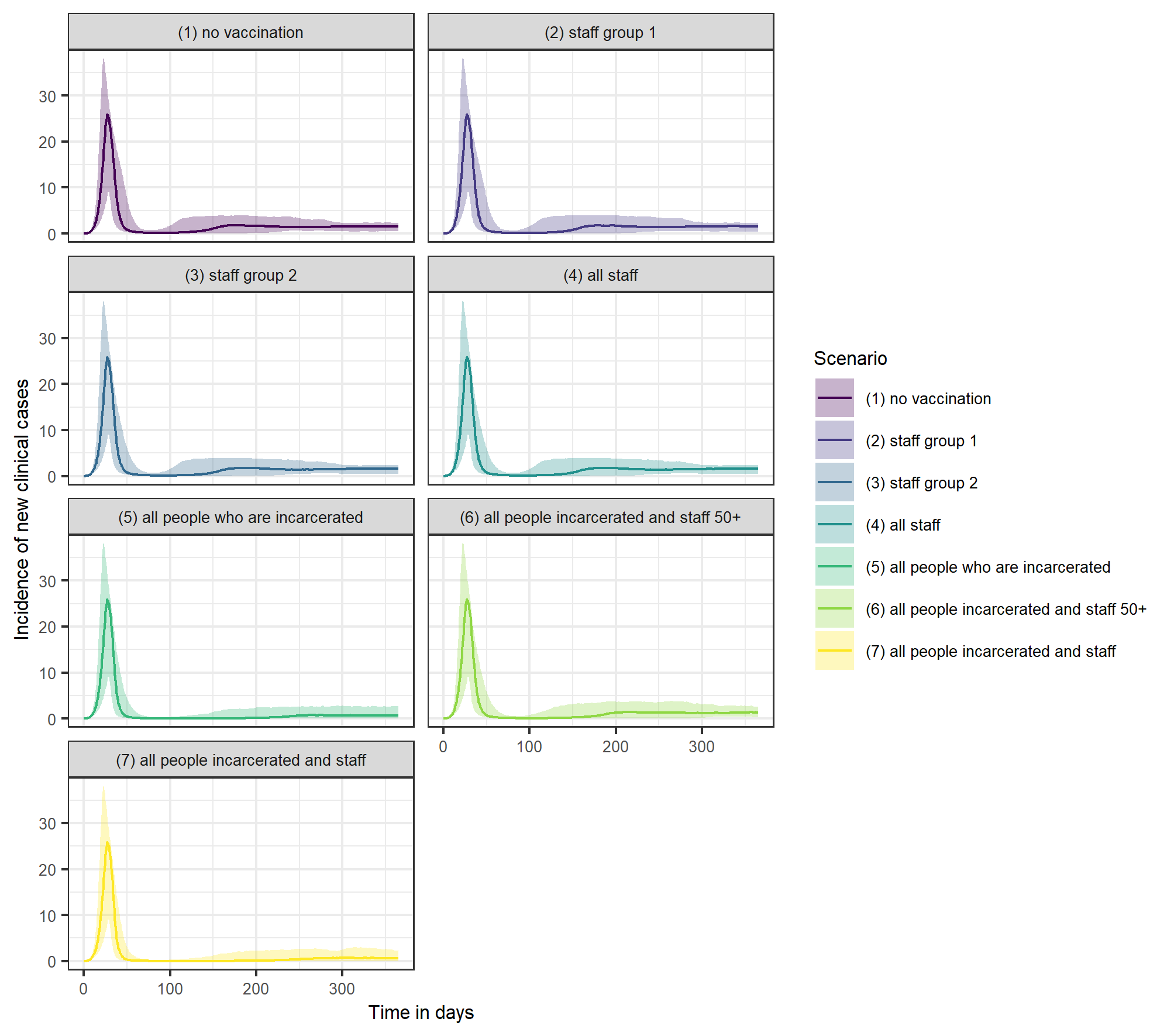


Supplementary Figure 2. Incidence of new clinical cases over time under each vaccination scenario, if vaccination is introduced at the start of an outbreak and administered at a rate of 50 doses per day.


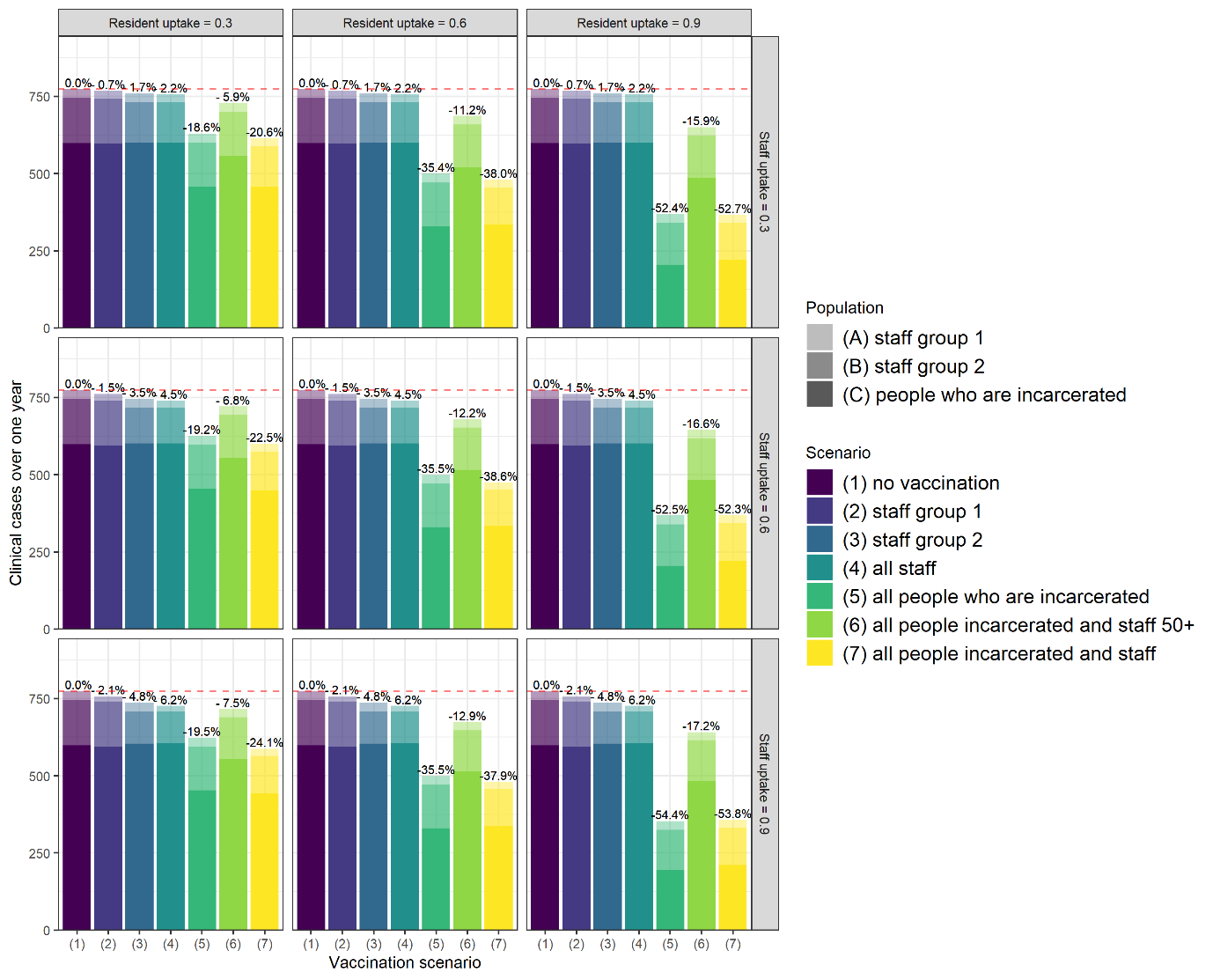


Supplementary Figure 3. Independently varying vaccine uptake between 30% and 90% among staff and individuals who are incarcerated.


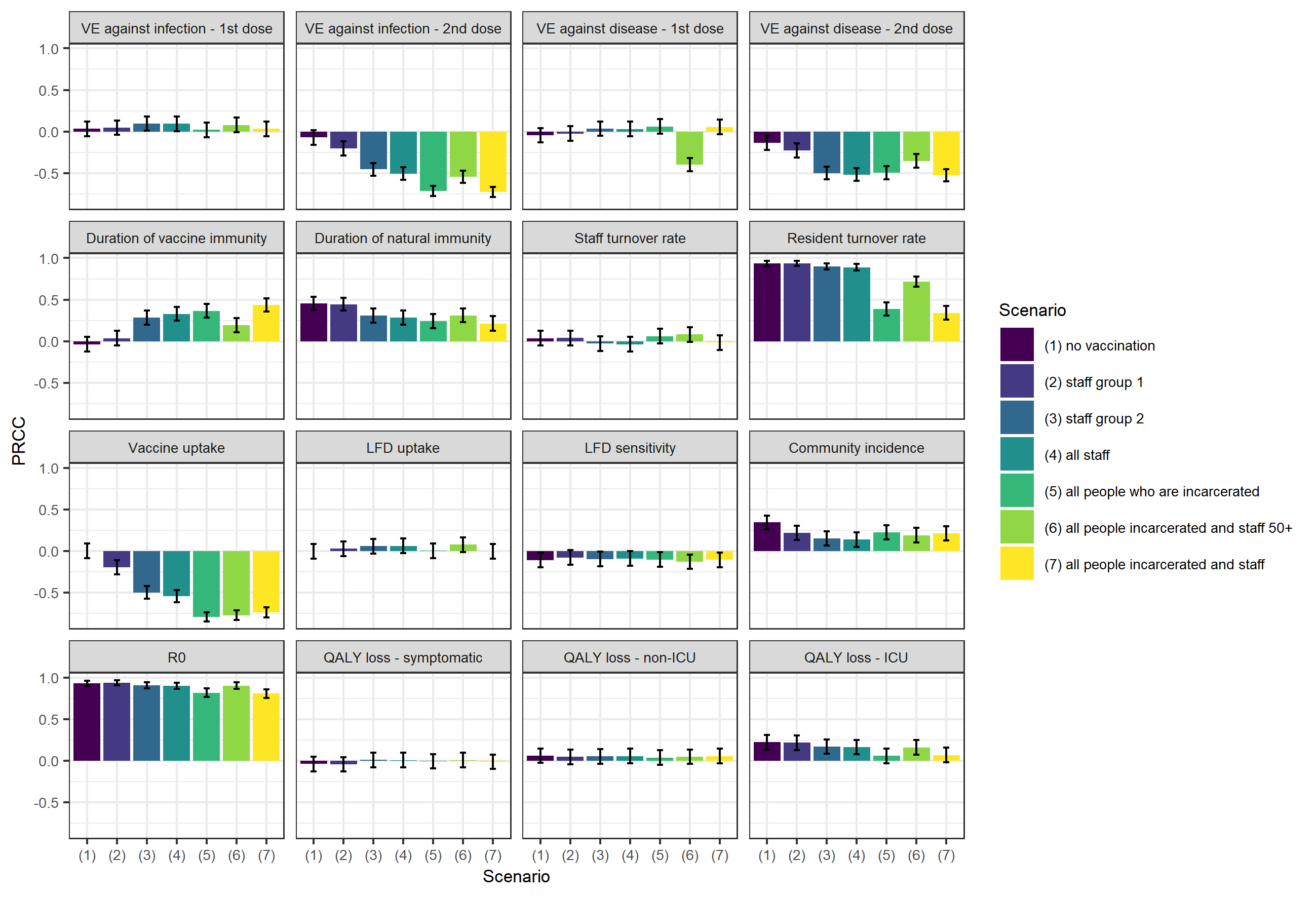


Supplementary Figure 4. Sensitivity of estimated total QALY loss averted under each vaccination scenario to variation in parameters.


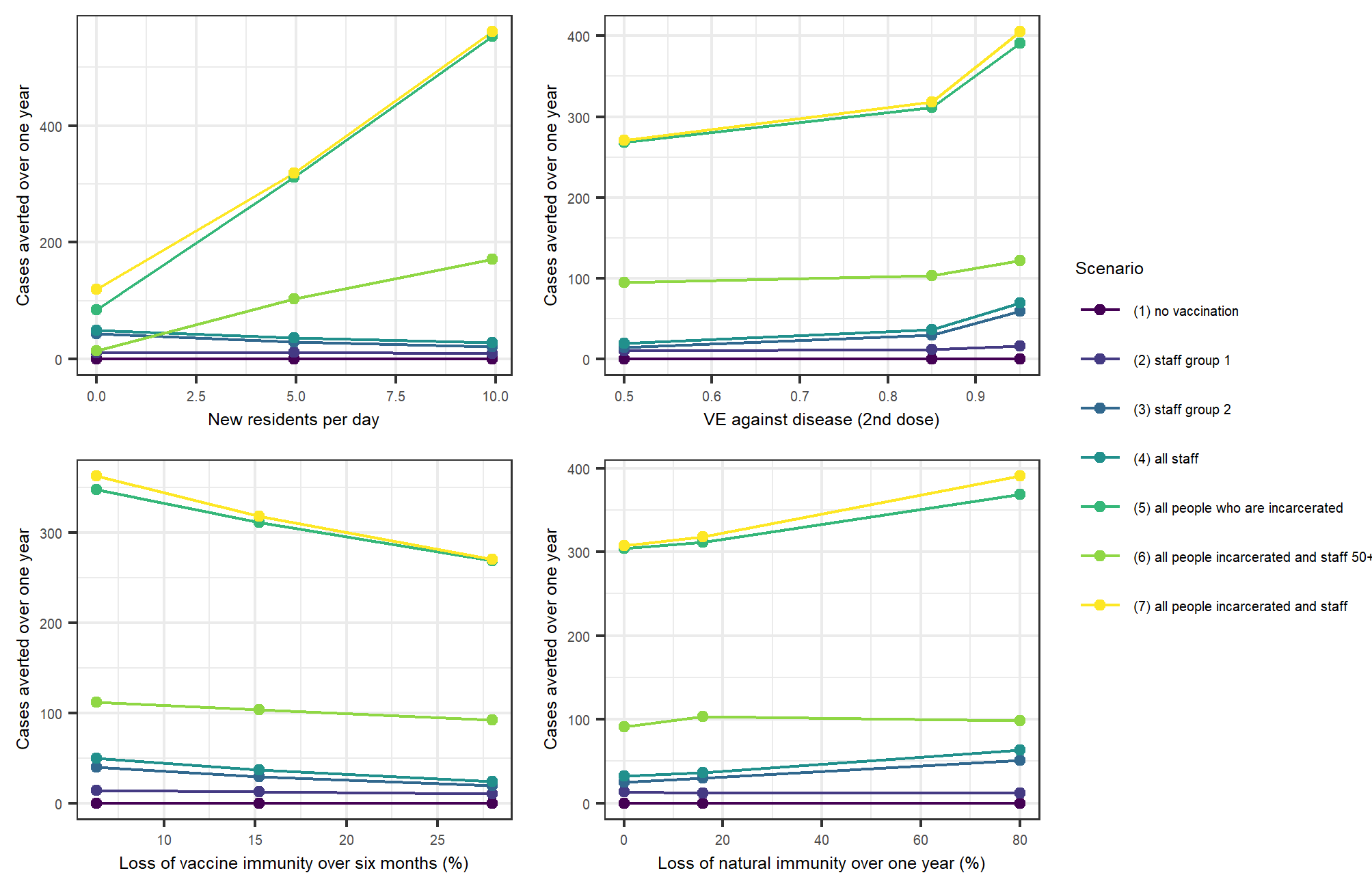


Supplementary Figure 5. Change in cases averted over one year with change in resident turnover, efficacy against disease (2nd dose), duration of vaccine immunity and duration of natural immunity.


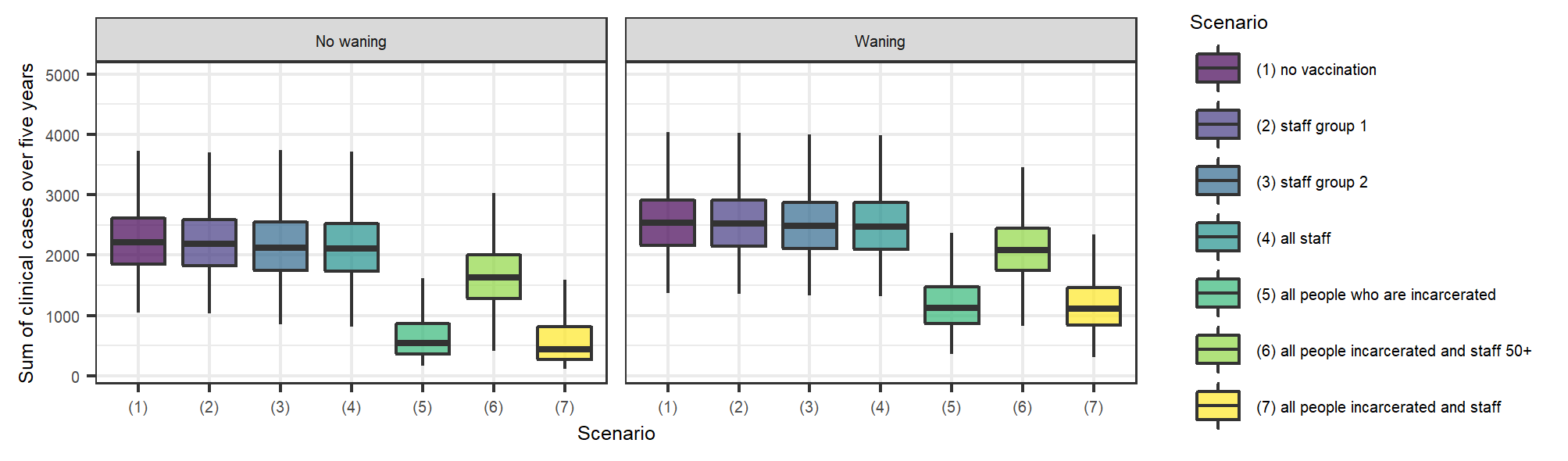


Supplementary Figure 6. Sum of clinical cases over five years when vaccine and natural immunity are assumed not to wane vs. waning of natural immunity of 16% over one year and waning of vaccine immunity of 19% over six months.


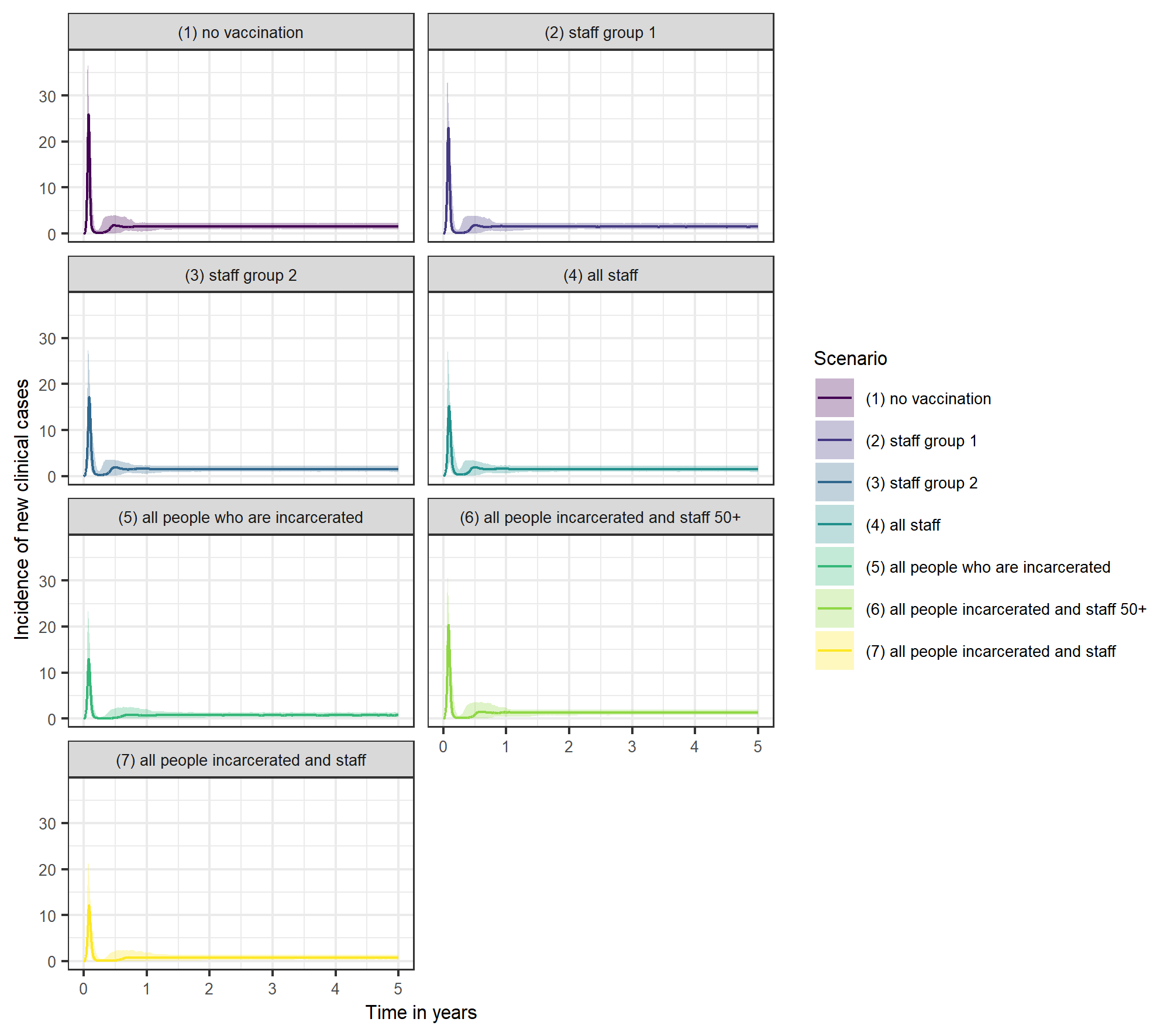


Supplementary Figure 7. Incidence of new clinical cases over five years under each vaccination scenario, including uncertainty captured using probabilistic sensitivity analysis.


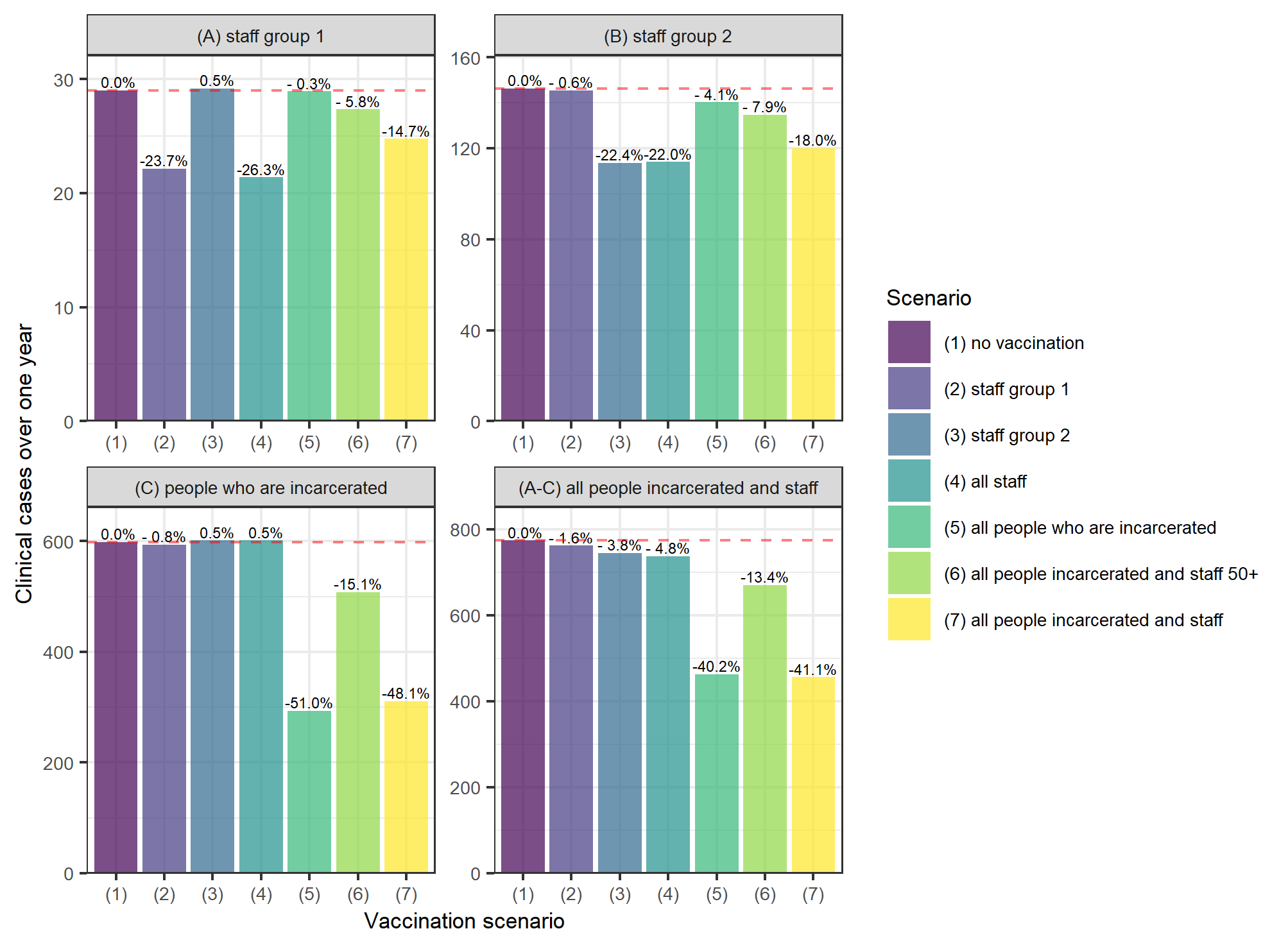


Supplementary Figure 8. Cases over one year in an average local male prison, by sub-population, under each of seven vaccination scenarios.


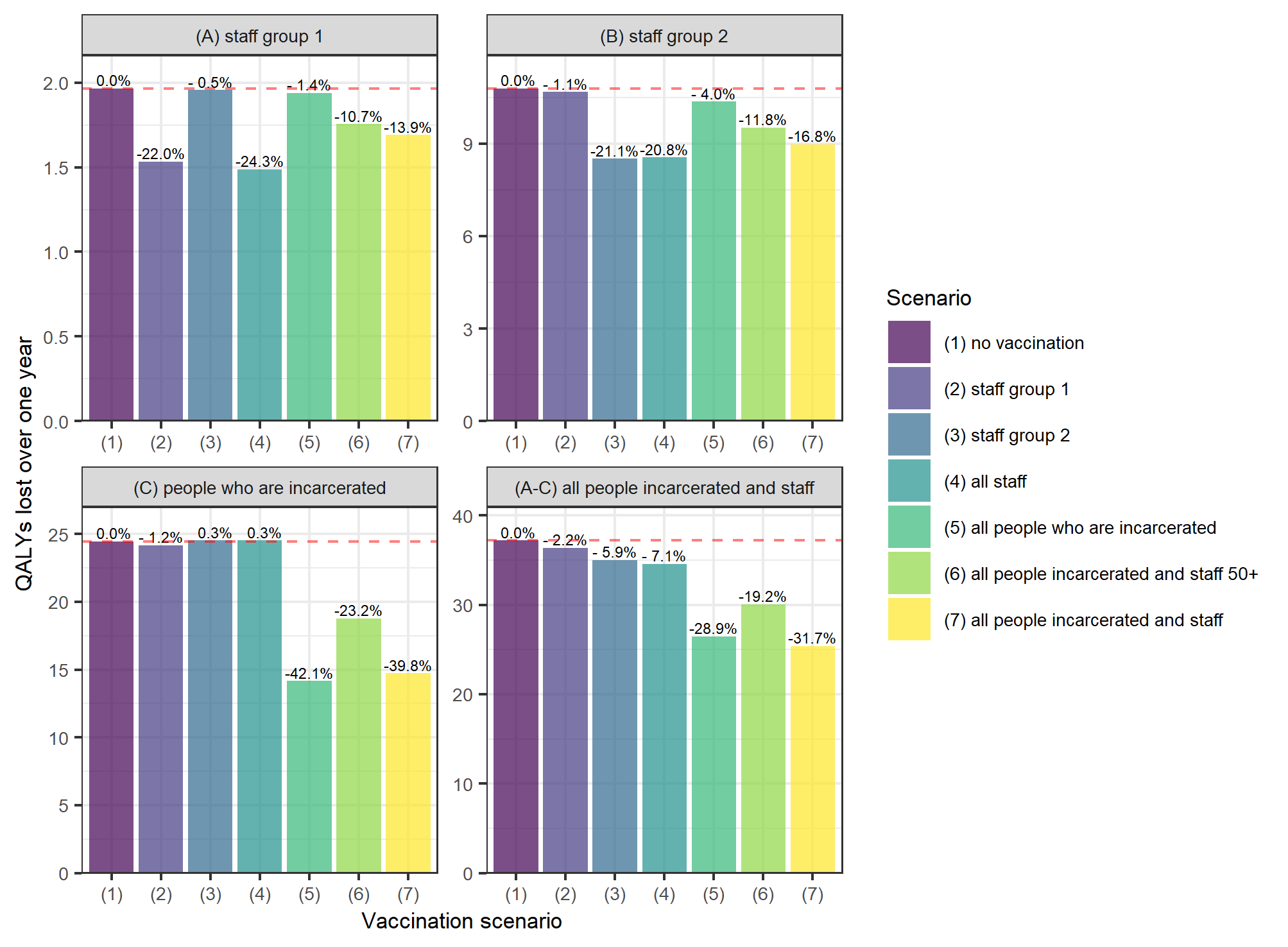


Supplementary Figure 9. QALY loss over one year in an average local male prison, by sub-population, under each of seven vaccination scenarios.


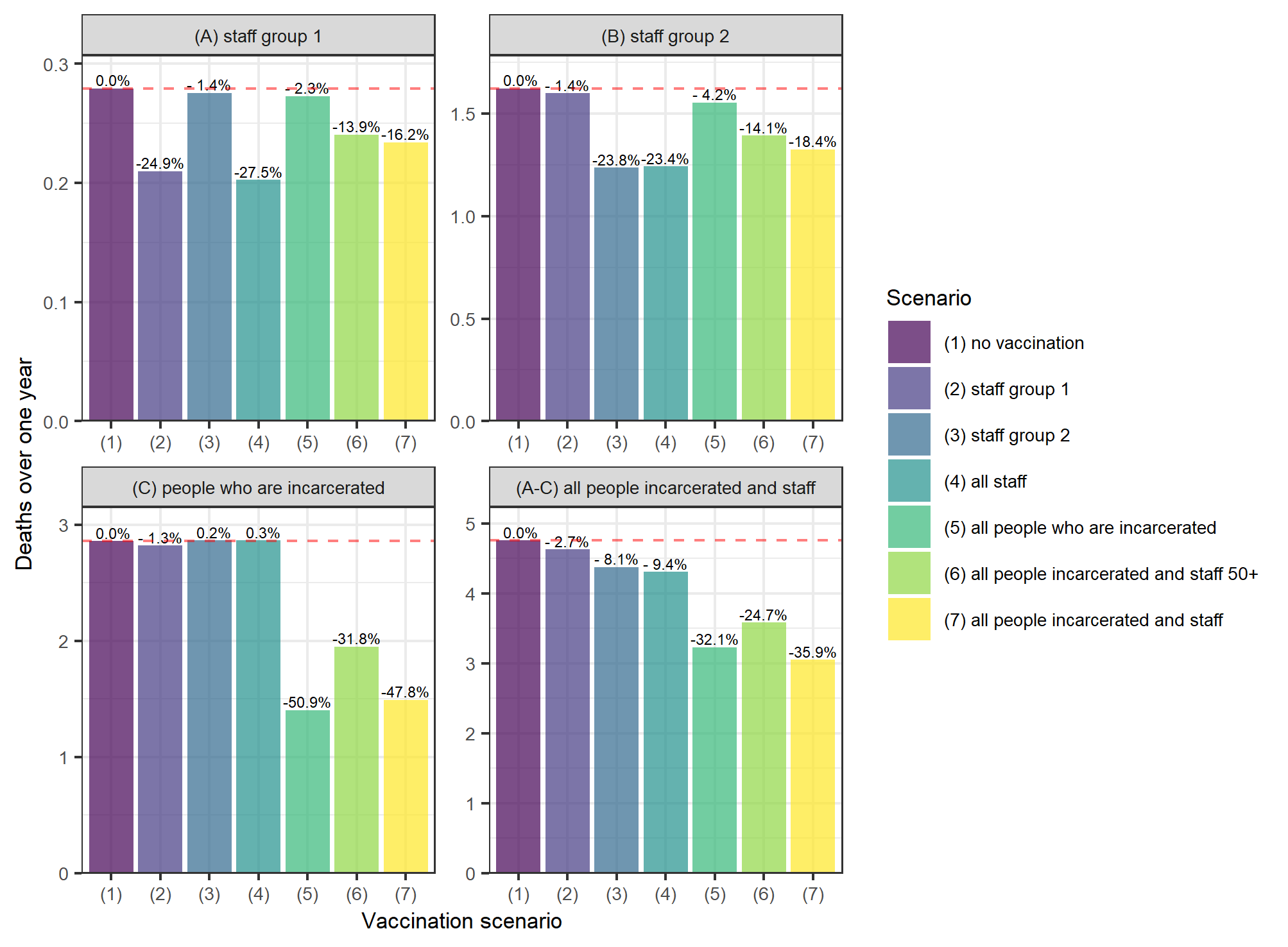


Supplementary Figure 10. Deaths over one year in an average male prison, by sub-population, under each of seven vaccination scenarios.

When cases, QALY loss and deaths are broken down by sub-population, some strategies result in increased health burden in the sub-populations that are unvaccinated. This likely results from the high population turnover: if the force of infection is decreased in the vaccinated population leading a lower but broader peak, this leads to a longer time period over which infected individuals from the vaccinated sub-population(s) are interacting with the unvaccinated sub-population(s). As these unvaccinated sub-populations are constantly replenished with new susceptible individuals, this leads to a higher number of cases overall. This phenomenon is not observed if population turnover is assumed to be zero in all sub-populations. This is driven in part by the way the model is set up, given that it is assumed that all new residents and staff are either susceptible or vaccinated, but not recovered. However, it remained the case if population turnover was lower, but not zero – which could be a proxy for assuming that only a certain proportion of new residents or staff are susceptible.

1. Davies NG, Klepac P, Liu Y, Prem K, Jit M, Eggo RM. Age-dependent effects in the transmission and control of COVID-19 epidemics. Nat Med. 2020 Aug;26(8):1205–11.

2. Comparative Analysis of the Risks of Hospitalisation and Death Associated with SARS-CoV-2 Omicron (B.1.1.529) and Delta (B.1.617.2) Variants in England by Tommy Nyberg, Neil M. Ferguson, Sophie G. Nash, Harriet H. Webster, Seth Flaxman, Nick Andrews, Wes Hinsley, Jamie Lopez Bernal, Meaghan Kall, Samir Bhatt, Paula Bianca Blomquist, Asad Zaidi, Erik Volz, Nurin Abdul Aziz, Katie Harman, Russell Hope, Andre Charlett, Meera A. Chand, Azra Ghani, Shaun Seaman, Gavin Dabrera, Daniela DeAngelis, Anne M. Presanis, Simon Thelwall :: SSRN [Internet]. [cited 2022 Mar 14]. Available from: https://papers.ssrn.com/sol3/papers.cfm?abstract_id=4025932

3. Office for National Statistics. Principal projection - England and Wales population in age groups [Internet]. 2022 [cited 2022 Mar 1]. Available from: https://www.ons.gov.uk/peoplepopulationandcommunity/populationandmigration/populationprojections/datasets/tablea23principalprojectionenglandandwalespopulationinagegroups

4. Docherty AB, Harrison EM, Green CA, Hardwick HE, Pius R, Norman L, et al. Features of 20 133 UK patients in hospital with covid-19 using the ISARIC WHO Clinical Characterisation Protocol: prospective observational cohort study. BMJ [Internet]. 2020;369. Available from: https://www.bmj.com/content/369/bmj.m1985

5. Briggs AH, Goldstein DA, Kirwin E, Meacock R, Pandya A, Vanness DJ, et al. Estimating (quality-adjusted) life-year losses associated with deaths: With application to COVID-19. Health Econ. 2021;30(3):699–707.

6. Graham L, Fischbacher CM, Stockton D, Fraser A, Fleming M, Greig K. Understanding extreme mortality among prisoners: a national cohort study in Scotland using data linkage. Eur J Public Health. 2015 Oct 1;25(5):879–85.

7. Jia H, Zack MM, Thompson WW. The Effects of Diabetes, Hypertension, Asthma, Heart Disease, and Stroke on Quality-Adjusted Life Expectancy. Value Health J Int Soc Pharmacoeconomics Outcomes Res. 2013;16(1):140–7.
